# Data Mining of Groundwater Genomes for Metagenome-Assembled Genomes (MAGs) Containing Monooxygenase Operons Associated with Contaminant Biodegradation

**DOI:** 10.3390/biology15181586

**Published:** 2026-09-09

**Authors:** Alison M. Cupples, James Richards, Maria Basaldua Del Cid

**Affiliations:** Department of Civil and Environmental Engineering, Michigan State University, East Lansing, MI 48824, USA; rich1261@msu.edu (J.R.); basaldua@msu.edu (M.B.D.C.)

**Keywords:** particulate methane monooxygenase, soluble methane monooxygenase, toluene monooxygenase, phenol monooxygenase, propane monooxygenase

## Abstract

This study examined freely available whole genome sequencing (WGS) data for genes associated with contaminant biodegradation. Thirteen WGS datasets (>600 individual samples) involving more than 12,000 Gbases from multiple countries were examined. The Department of Energy Systems Biology Knowledgebase (KBase) was used to create metagenome-assembled genomes (MAGs) containing the operons of interest. The arrangement and length of subunits for each operon were compared. Phylogenetic trees were created for common biomarkers (*tmoA*, *pmoA*, *prmA*, *mmoX*, *dmpN*). MAGs were uploaded into publicly available KBase narratives. Thirty-four MAGs, within the phyla *Actinomycetota*, *Pseudomonadota* and *Chloroflexota*, were identified with the full propane monooxygenase operon (*prmABCD*). Twenty-six MAGs, within the classes *Gammaproteobacteria* and *Alphaproteobacteria*, contained the full operon for soluble methane monooxygenase (*mmoXYBZDC*). More than 100 MAGs contained the full operon for ammonia/particulate methane monooxygenase (*pmoCAB*) and were classified within the *Gammaproteobacteria* and *Alphaproteobacteria* groups, as well as other phyla. Fifty-five MAGs, within *Burkholderiales* (*Gammaproteobacteria*) and *Alphaproteobacteria*, contained the full operon for toluene-4-monooxygenase (*tmoABCDEF*). From the MAGs containing the full operon for toluene monooxygenase, thirty-three also contained the full operon for phenol monooxygenase (*dmpKLMNOP*). The MAGs generated and their associated functional gene sequences have the potential to improve molecular detection methods for site bioremediation.

## Simple Summary

Environmental contaminants are often degraded by bacteria already present in soil and water. The occurrence of particularly active bacteria can be investigated by targeting specific nucleic acid (DNA) sequences. The objective of the current study was to investigate freely available nucleic acid sequences obtained from numerous previous studies across multiple countries to determine which species of active bacteria are common and which active genes they contain. For this, the Department of Energy Systems Biology Knowledgebase (KBase) was used to analyze the raw data and create assembled genomes for each microorganism of interest. The sequences of numerous active genes were compared between these assembled genomes. These sequences were then made available to the public through KBase websites. The most commonly detected active genes included those encoding for the enzymes methane monooxygenase, propane monooxygenase, phenol monooxygenase and toluene monooxygenase. The information will be particularly useful to environmental engineers involved in the design of molecular assays for the quantification of active bacteria in contaminated groundwater and soils.

## 1. Introduction

A promising approach for site bioremediation is the use of aerobic metabolism and co-metabolism because these processes can degrade multiple contaminants, and there is the potential to degrade these contaminants to low levels [1,2,3,4,5,6,7,8,9]. A number of oxygenases and microorganisms have been associated with such detoxification processes. In particular, a large amount of evidence exists for the co-metabolic biodegradation of contaminants comprising methane, propane, toluene or phenol monooxygenases. As bioremediation often involves multiple lines of evidence for proof of effective biodegradation, engineers frequently monitor the occurrence of the genes encoding for these enzymes at contaminated sites. To optimize these monitoring approaches, it is important to understand the diversity associated with these biomarkers across groundwater metagenomes.

Early research on contaminant co-metabolism focused on methanotrophs, microorganisms that use methane as a sole carbon and energy source. These microorganisms contain methane monooxygenase (MMO), an enzyme associated with the oxidation of methane to methanol. This enzyme is occurs in a soluble cytoplasmic form (sMMO) or a particulate membrane-associated form (pMMO) [10]. Particulate methane monooxygenase has been detected in all methanotrophs, except for in the genera *Methylocella* [11] and *Methyloferula* [12], whereas sMMO has been found in fewer strains [13]. The substrate range for sMMO includes monoaromatics, diaromatics, alkanes, alicyclic hydrocarbons, alkenes, and substituted methane derivatives [10]. Particulate methane monooxygenase is similar to ammonia monooxygenase from ammonia-oxidizing bacteria [14,15] and has been linked to the degradation of TCe and short linear hydrocarbons [10]. Propane monooxygenase is another enzyme important for the removal of environmental pollutants. For example, indigenous microorganisms degraded 1,4-dioxane, TCE and 1,2-dichloroethane when propane and oxygen were added to groundwater [16]. Other co-metabolic contaminant degraders include microorganisms associated with toluene oxidation [17,18,19,20]. For example, toluene-4-monooxygenase (encoded by *tmoABCDEF)* [21,22,23] has been linked with the degradation of N-nitrosodimethylamine; polycyclic aromatic substrates; cDCE; TCE; 1,1-DCE; 1,4-dioxane; alkenes; chloroform and substituted benzenes [22,23,24,25,26,27,28,29,30,31]. Catechol and substituted catechols are intermediate chemicals in the biodegradation of many pollutants [32,33,34]. For example, phenol monooxygenase from *Pseudomonas* sp. strain CF600 is important for the biodegradation of phenol to catechol [35].

Knowledge regarding the occurrence of microorganisms with such degradative capabilities should enhance our understanding of the removal of many contaminants. Typically, quantitative PCR (qPCR) is used to determine the presence and abundance of these functional genes in environmental samples. However, these assays are frequently based on pure cultures sequences and often do not take into account the gene sequences actually present in groundwater or subsurface sediments. For this evaluation, the current study examined freely available whole genome sequencing (WGS) data to determine gene sequences actually found in environmental groundwater. In the past decade, WGS has exploded as an investigative tool for mixed microbial communities across a large number of disciplines. However, the use of this method in environmental engineering to examine bioremediation potential at contaminated sites is still in its infancy, primarily due to (1) sequencing costs and (2) challenges relating to data analysis. The development of an on-line sequencing analysis platform, the Department of Energy Systems Biology Knowledgebase, or KBase [36]., has the potential to remove one of these limitations. KBase is free, easy to use, sharable across users and offers fully developed tools (e.g., quality control, annotation, creation of metagenome-assembled genomes) to analyze WGS datasets. The increase in publicly available WGS data from environmental samples from the National Center for Biotechnology Information (NCBI) Sequence Read Archive (SRA) database has the potential to remove the other limitation. There are several hundreds of gigabytes from WGS runs from environmental samples, and these datasets have yet to be tapped as a resource for understanding contaminant biodegradation potential.

The current project involved several objectives. One objective was to collect and analyze freely available groundwater sequencing data (WGS datasets) for the functional genes (listed above) previously associated with contaminant biodegradation from multiple studies across multiple countries. A second objective was to summarize the phylotypes associated with these functional genes. A third objective was to compare the gene sequences of the subunits commonly used as biomarkers for each functional gene (*tmoA*, *pmoA*, *prmA*, *mmoX*, *dmpN*) and, when applicable, to identify patterns for each di-iron center. A final objective was to make the resulting data freely available so others can update genetic detection methods for these key genes. For this, metagenome-assembled genomes (MAGs), with full operons for the targeted genes, were collected and made available in a group of KBase narratives.

## 2. Methods

### 2.1. Selection of Publicly Available Sequencing Data

Thirteen WGS datasets, with more than 600 individual samples, involving more than 12,000 Gbases, were downloaded from previous studies focusing on the molecular analysis of groundwater (Table 1). The datasets originated from nine different countries (USA, New Zealand, Japan, Canada, Sweden, China, Bangladesh, Germany and The Netherlands). The datasets were selected based on the size of data for each sample (i.e., larger files), as preliminary data analysis with smaller files did not generate MAGs with the desired genes. To obtain the datasets, NCBI’s SRA database was searched using keywords such as “groundwater” and “shotgun sequencing” or “WGS”. The results were analyzed further to exclude those that were not relevant (e.g., amplicon sequencing data, non-typical groundwater). The datasets were downloaded to the High Performance Computing Cluster (HPCC) at MSU using the SRA toolkit, as most files were >5 GB. The International Nucleotide Sequence Database Collaboration policy allows unrestricted access to all data records to enable publication, with appropriate credit being given by citing the original submission (accession numbers). The final datasets were obtained primarily from groundwater studies (pristine as well as contaminated sites), with a range of samples (from 6 to >300) and a total number of bases. However, we recognize that microorganisms in pristine environments may not survive at contaminated sites. Additional information for each dataset and study can be found in the references listed (Table 1).

### 2.2. Analysis of Whole Genome Sequencing Data

The WGS files were uploaded from HPCC to the United States Department of Energy Systems Biology Knowledgebase (KBase) [36] using Globus v3.3.0 (freely available at MSU). Due to the large amount of data, more than eighty narratives were generated. For example, the data collected from BioProject Accession number PRJNA858913 was analyzed in forty narratives. The steps involved for the analysis of each narrative were the same. The sequencing files were first subjected to quality control and filtering using FastQC (version 0.12.1) [53] as well as Trimmomatic (version 0.36) (sliding window size of 4; sliding window minimum quality of 15) [54]. Megahit (version 1.2.9) [55] was then used to assemble the read libraries using 2000 bp as a minimum read length. RASTtk (version 1.073) [56] and Prokka (version 1.14.16) [57] were then used to annotate the read libraries. The occurrence of each target gene in each assembly was determined using the search function in KBase, e.g., searching for “methane” or “toluene”.

For metagenomes containing the target genes, the contigs were binned using three binning approaches: MaxBin2 (version 2.2.4) [58], CONCOCT (version 1.1) [59], and MetaBAT2 (version 1.7) [60]. The bins from the three approaches were then optimized using Das Tool (version 1.1.2) [61]. Then, CheckM (version 1.0.18) [62] was used to filter the bins for quality. The KBase app called Extract Bins as Assemblies from BinnedContigs (version 1.0.2) [36] was used to extract the bins that passed the quality filtering step. The KBase app Annotate Multiple Microbial Assemblies with RASTtk (version 1.073) [56] was used to annotate the extracted bins. Finally, the KBase app called Classify Microbes with GTDB-Tk (version 1.7.0) [63] was used to determine the taxonomy of each extracted bin. Only MAGs with completeness ≥ 80% and contamination ≤ 20% were included.

### 2.3. Selection of Metagenome-Assembled Genomes

The annotated bins, also called MAGs, were examined for the presence of the target genes using the KBase search function. When present, the subunit sequence representing key biomarkers for each gene (e.g., *tmoA*, *pmoA*, *prmA*, *mmoX)* were collected and stored in text files. Only MAGs containing all subunits (of the correct length and orientation) of each target gene on the same contig were selected for downloading. This involved four subunits for propane monooxygenase (*prmABCD*), including propane monooxygenase large subunit (*prmA*), reductase subunit (*prmB*), small subunit (*prmC*) and coupling protein (*prmD*) [64]. Soluble methane monooxygenase (sMMO) involves a six-gene operon (*mmoXYBZDC*), encoding for the α, β, and γ subunits of the hydroxylase (*mmoXYZ*), reductase (*mmoC*) and a regulatory or coupling protein (*mmoB*) [10]. MMOD is a regulatory entity to repress expression of sMMO [65]. Particulate MMO (pMMO) is encoded by the *pmoCAB* operon [65], which shares similarities with ammonia monooxygenase (AMO, encoded by *amoCAB*) resulting from ammonia-oxidizing bacteria [14,15].

Toluene-4-monooxygenase is encoded by the gene cluster *tmoABCDEF* [21,22,23]. The genes *tmoA*, *tmoB*, and *tmoE* encode the α, β, and γ subunits, respectively, that form the (αβγ)_2_ quaternary structure of the hydroxylase component [66]. The genes *tmoC*, *tmoD*, and *tmoF* encode the [2Fe-2S] ferredoxin, the effector protein, and the NADH oxidoreductase, respectively [66,67,68,69]. All MAGs containing the target genes were downloaded from individual narratives, then uploaded to publicly available KBase narratives under the following names: “Data Mining Propane Monooxygenase”, “Data Mining Soluble Methane Monooxygenase”, “Data Mining Particulate Methane/Ammonia Monooxygenase Burkholderiales Pseudomonadales and Nevskiales”, “Data Mining Particulate Methane/Ammonia Monooxygenase Methylococcales”, “Data Mining Particulate Methane/Ammonia Monooxygenase Other Phyla” and “Data Mining Toluene Monooxygenase”. The MAGs containing particulate methane/ammonia monooxygenase operons were uploaded to three separate narratives due to the large number of MAGs obtained. Links to all KBase narratives containing all MAGs are available at the following narrative “Data Mining of Groundwater to Identify MAGs with Methane, Propane and Toluene Monooxygenases” (https://narrative.kbase.us/narrative/242870, accessed on 1 July 2026).

The toluene monooxygenase containing MAGs were also examined for the presence of phenol monooxygenase, as recent studies documented the co-occurrence of toluene monooxygenase and phenol monooxygenase in *Rhodoferax* and *Azonexus* MAGs [70,71]. The gene cluster *dmpKLMNOP* encodes for phenol monooxygenase [35]. DmpL-DmpN-DmpO form the oxygenating component of the hydroxylase. DmpN contains the dinuclear iron center and the active site [72,73,74]. The enzyme also has a reductase component, containing one iron–sulfur cluster and FAD (DmpP), and a regulatory component (DmpM) [74,75,76,77]. DmpK is important for the iron-dependent assembly of an active oxygenase [76]. Transcription is positively regulated by the product of *dmpR*, with expression occurring only when the substrates are present [78,79,80].

### 2.4. Analysis of Metagenome-Assembled Genomes and Genes

Phylogenetic information and the source of each MAG containing the genes of interest were summarized across all samples. As stated above, when present, the subunit sequence representing key biomarkers for each gene (e.g., *tmoA*, *pmoA*, *prmA*, *mmoX*, *dmpN)* were collected, stored in text files, and used to generate phylogenetic trees using MEGA 12 [81]. Due to the large number of sequences collected for *pmoA*, only one representative sequence for each phylotype was used for each tree. The subunit size and orientations were illustrated in gene arrow plots created with R (version 4.2.1) [82] in RStudio (version 2022.12.0) [83] and the R packages readxl (version 1.4.2) [84], ggplot2 (version 3.4.4) [85], and gggenes (version 0.5.1.) [86]. Due to the large number of sequences obtained, the gene arrow diagrams were divided into groups according to their phylogenetic classification.

## 3. Results

### 3.1. Phylogenetic Classification of MAGs with the Target Operons

Tables were generated to summarize the classification of the MAGs containing the targeted operons (Supplementary Tables S1–S10). Each table contains bin and SRR numbers that correspond to the MAGs in the publicly available KBase narratives (see below). In all, thirty-four MAGs, obtained from five different datasets (different BioProject Accession Numbers), were identified as containing the full propane monooxygenase operon (*prmABCD*). From these, eighteen were classified with the phylum *Actinomycetota* (Supplementary Table S1) and sixteen with the phyla *Pseudomonadota* and *Chloroflexota* (Supplementary Table S2). The majority of those within the phylum *Actinomycetota* were in the order *Mycobacteriales* and family *Mycobacteriaceae* (*Rhodococcus*, *Mycobacterium*, *Williamsia* and *Gordonia*). The remaining *Actinomycetota* MAGs were in the orders *Rubrobacterales* and *Solirubacterales*. MAGs classifying within the *Pseudomonadota* belonged to the orders *Rhizobiales*, *Acetobacterales*, *Rhodobacterales*, *Burkholderiales* and *Ktedonobacterales*.

Twenty-six MAGs (from six datasets) contained the full operon for soluble methane monooxygenase (*mmoXYBZDC*) (Supplementary Table S3). The majority (twenty-four) classified within the class *Gammaproteobacteria*, and the remaining two classified within the class *Alphaproteobacteria.* Notably, the MAGs classified within only three families (*Methylomonadaceae*, *Methylophilaceae* and *Beijerinckiaceae*). At the genus level, sMMO containing MAGs classifying as *Methylobacter*, *Methylomonas* and *Methylocella* were identified multiple times.

A large number of MAGs (>100), from nine datasets, contained the full operon for ammonia/particulate methane monooxygenase (Supplementary Tables S4–S6). Forty-seven classified within the order *Methylococcales* (*Gammaproteobacteria*), with the majority of these belonging to the family *Methylomonadaceae*, and commonly detected genera including *Methylobacter*, *Methylicorpusculum*, *Methylovulum*, *Methylomonas*, and *Methyloglobulus* (Supplementary Table S4). Thirty-two classified within the orders *Burkholderiales* and *Pseudomonadales* and *Nevskiales* (*Gammaproteobacteria*) (Supplementary Table S5). The remaining MAGs containing this operon classified with class *Alphaproteobacteria* (e.g., *Methylosinus*, *Methylocystis*, *Methylocella*) and phyla *Methylomirabilota*, *Bacteroidota*, *CSP1-3*, *Nitrospirota*, *Actinomycetota* and *Desulfobacterota_B* (Supplementary Table S6).

Fifty-five MAGs (from ten datasets) contained the full operon for toluene monooxygenase (Supplementary Tables S7 and S8). From these, thirty classified within the *Burkholderiaceae* (*Burkholderiales*, *Gammaproteobacteria*) (Supplementary Table S7), twenty-four classified within the *Rhodocyclaceae* (*Burkholderiales*, *Gammaproteobacteria*) (Supplementary Table S8), and two classified within the *Alphaproteobacteria* (Supplementary Table S8). The majority of MAGs classified in a small number of genera (*Azonexus*, *Hydrogenophaga*, *Rhodoferax* and *Trinickia*). Interestingly, from the MAGs containing the full operon for toluene monooxygenase, thirty-three (*Azonexus*, *Hydrogenophaga*, *Rhodoferax*, *Trinickia*, *Sphaerotilus*, *Polaromonas*, *Ramlibacter*, *Limnobacter*, *Zoogloea*) also contained the full operon for phenol monooxygenase (Supplementary Tables S9 and S10).

### 3.2. Operon Characterization and Phylogenetic Trees

**Figure 1:**
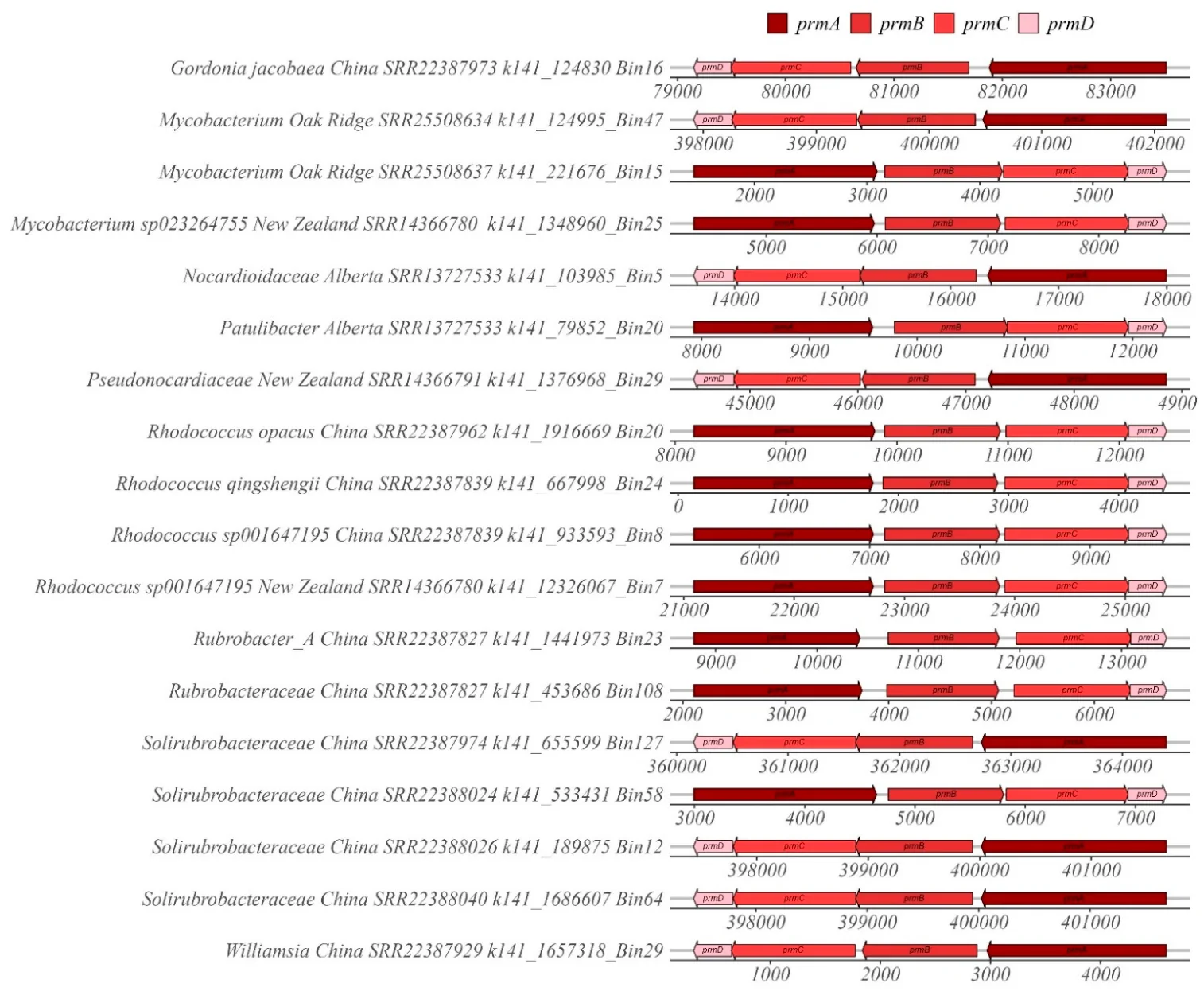
Subunit length and arrangement of propane monooxygenase operons in groundwater metagenomes associated with the identified metagenome-assembled genomes within the phylum *Actinomycetota*.

**Figure 2:**
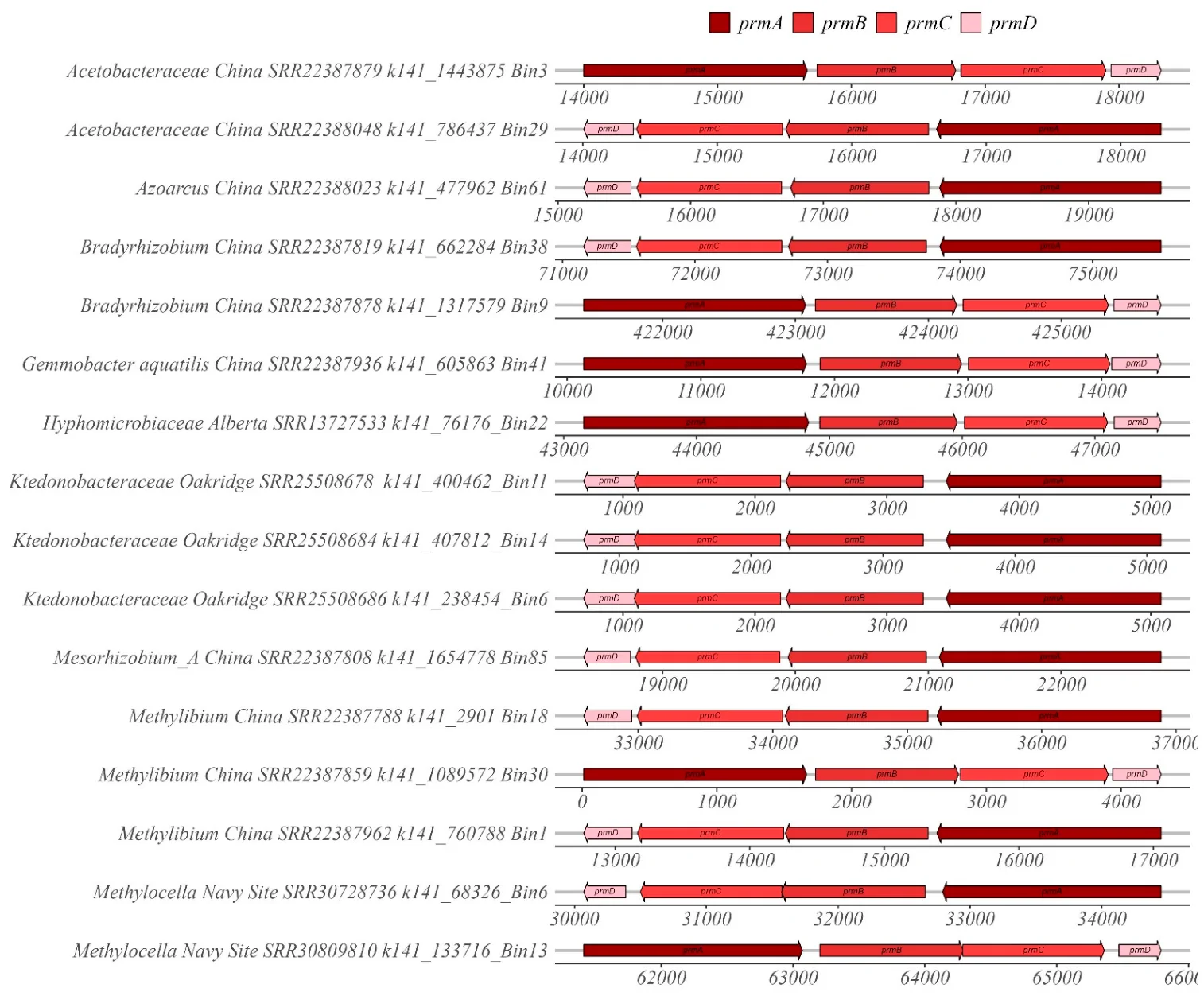
Subunit length and arrangement of propane monooxygenase operons in groundwater metagenomes associated with the identified metagenome-assembled genomes within the phyla *Pseudomonadota* and *Chloroflexota*.

The size and alignment of each subunit was determined for each operon of interest obtained from the sequencing data. In general, the subunit lengths and orientation were similar to those previously reported (NCBI) and determined in our previous studies [9,71,87,88,89]. MAGs containing the four subunits of the propane monooxygenase operon (*prmABCD*) classified within the phyla *Actinomycetota*, *Pseudomonadota* and *Chloroflexota* (Figure 1 and Figure 2). For operons within MAGs classifying as phylum *Actinomycetota*, the average ± standard deviation (range) for the number of bases for *prmA*, *prmB*, *prmC* and *prmD* was 1643 ± 14 (1628–1664), 1053 ± 17 (1043–1097), 1120 ± 21 (1100–1169), and 350 ± 10 (341–374), respectively (Figure 1). Similarly, for operons within MAGs classifying within the phyla *Pseudomonadota* and *Chloroflexota*, the average ± standard deviation (range) for the number of bases for *prmA*, *prmB*, *prmC* and *prmD* was 1668 ± 9 (1655–1694), 1057 ± 18 (1034–1088), 1083 ± 11 (1058–1097), and 354 ± 16 (320–374), respectively (Figure 2).

MAGs containing the soluble methane monooxygenase operon (*mmoXYBZDC*) classified within the *Gammaproteobacteria* and *Alphaproteobacteria* (Supplementary Figures S1 and S2). For operons within MAGs classifying within the *Gammaproteobacteria* and *Alphaproteobacteria*, the average ± standard deviation (range) for the number of bases for *mmoX*, *mmoY*, *mmoB*, *mmoZ*, *mmoD* and *mmoC* was 1584 ± 6 (1577–1604), 1178 ± 1 (1175–1181), 424 ± 2 (419–425), 499 ± 4 (494–506), 264 ± 8 (242–269), and 1036 ± 2 (1031–1037), respectively (Supplementary Figure S1). Notably, eleven MAGs containing the soluble methane monooxygenase operon did not annotate the subunit D; however, the hypothetical gene was determined to be in the same position at the same length (Supplementary Figure S2). For these, the characteristics of the subunits were similar to those listed above, and the average ± standard deviation (range) for the number of bases for *mmoX*, *mmoY*, *mmoB*, *mmoZ*, *hypothetical* and *mmoC* was 1582 ± 2 (1580–1583), 1176 ± 2 (1175–1178), 417 ± 3 (413–425), 502 ± 6 (497–512), 274 ± 25 (245–311), and 1032 ± 23 (1037–1085), respectively.

Due to the large number of particulate ammonia/methane monooxygenase operons obtained, only one representative operon for each phylotype (in bold for Supplementary Tables S5–S7) was selected for determining subunit lengths and for generating the gene arrow figures (Supplementary Figures S3–S5). For operons within MAGs classifying within the order *Methylococcales* (*Gammaproteobacteria*) (Supplementary Table S4), the average ± standard deviation (range) for the number of bases for *pmoC*, *pmoA and pmoB* was 753 ± 5 (752–773), 744 ± 2 (743–749), and 1243 ± 3 (1232–1244), respectively (Supplementary Figure S3). For operons within MAGs classifying within the orders *Burkholderiales* and *Pseudomonadales* and *Nevskiales* (*Gammaproteobacteria*) (Supplementary Table S5) the average ± standard deviation (range) for the number of bases for *pmoC*, *pmoA and pmoB* was 787 ± 32 (710–851), 811 ± 22 (770–833), and 1270 ± 14 (1250–1292), respectively (Supplementary Figure S4). For operons within MAGs classifying within the class *Alphaproteobacteria* and phyla *Methylomirabilota*, *Bacteroidota*, *CSP1-3*, *Nitrospirota*, *Actinomycetota* and *Desulfobacterota_B* (Supplementary Table S6), the average ± standard deviation (range) for the number of bases for *pmoC*, *pmoA and pmoB* was 795 ± 37 (701–860), 779 ± 37 (725–830), and 1262 ± 24 (1214–1307), respectively (Supplementary Figure S5).

All MAGs containing the toluene monooxygenase operon classified within the order *Burkholderiales* (*Gammaproteobacteria*) (Supplementary Figures S6 and S7). For operons within MAGs classifying within the *Burkholderiaceae* (*Burkholderiales*, *Gammaproteobacteria*) (Supplementary Table S7), the average ± standard deviation (range) for the number of bases for *tmoA*, *tmoB*, *tmoC*, *tmoD*, *tmoE* and *tmoF* was 1499 ± 32 (1331–1505), 263 ± 3 (260–272), 355 ± 0, 315 ± 3 (308–326), 987 ± 3 (986–998), and 1021 ± 11 (1004–1037), respectively (Supplementary Figure S6). For MAGs classifying within the *Rhodocyclaceae* (*Burkholderiales*, *Gammaproteobacteria*) and *Alphaproteobacteria* (Supplementary Table S8), the average ± standard deviation (range) for the number of bases for *tmoA*, *tmoB*, *tmoC*, *tmoD*, *tmoE* and *tmoF* was 1505 ± 1 (1499–1508), 266 ± 5 (257–284), 335 ± 2 (335–344), 391 ± 62 (308–440), 986 ± 1 (986–989), and 1016 ± 10 (989–1046), respectively (Supplementary Figure S7).

For MAGS containing both operons for toluene monooxygenase and phenol monooxygenase, classifying within the *Azonexus*, the average ± standard deviation (range) for the number of bases for *2*,*3-catechol 2*,*3-dioxygenase*, *dmpK*, *dmpL*, *dpmM*, *dpmN*, *dpmO* and *dpmL* was 926 ± 0, 275 ± 0, 989 ± 0, 269 ± 0, 1553 ± 0, 389 ± 14 (359–395), and 1061 ± 0, respectively (Supplementary Figure S8). For all other MAGs (*Hydrogenophaga*, *Ramlibacter*, *Rhodoferax*, *Trinickia* and *Zoogloea*) with both operons, the average ± standard deviation (range) for the number of bases for *2*,*3-catechol 2*,*3-dioxygenase*, *dmpK*, *dmpL*, *dpmM*, *dpmN*, *dpmO* and *dpmL* was 937 ± 7 (926–944), 250 ± 22 (224–287), 994 ± 2 (992–998), 269 ± 0, 1544 ± 19 (1520–1562), 356 ± 1 (356–359), and 1062 ± 5 (1055–1073), respectively (Supplementary Figure S9). There were also nine MAGs that contained both operons in close proximity (Supplementary Figure S10).

**Figure 3:**
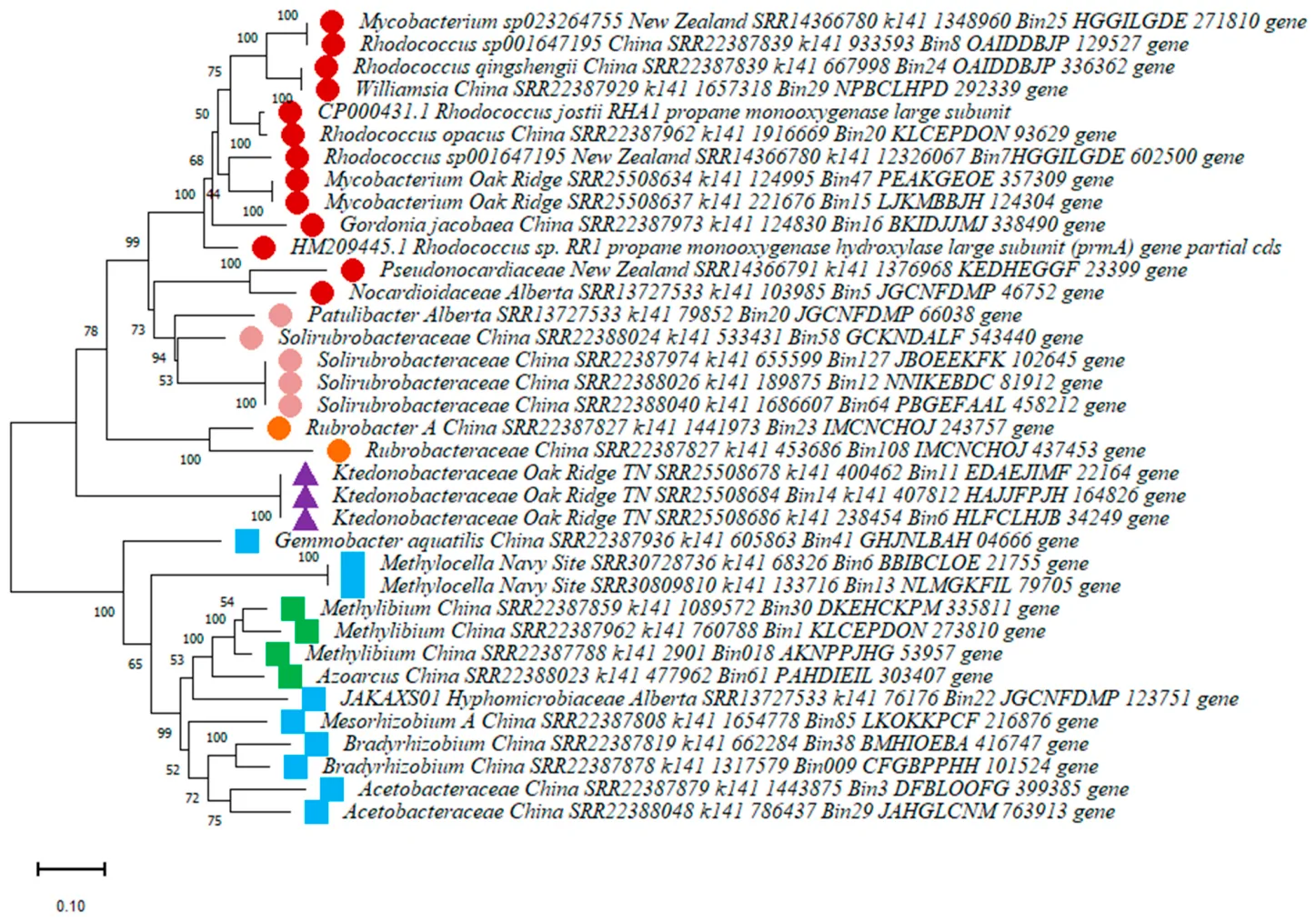
Alignment of *prmA* from MAGs containing the propane monooxygenase operon within the phyla *Actinomycetota* (class *Actinomycetia,* red circles; class *Rubrobacteria,* orange circles; class *Thermoleophilia,* pink circles), *Pseudomonadota* (class *Alphaproteobacteria,* blue squares; class *Gammaproteobacteria,* green squares) and *Chloroflexota* (purple triangles). The evolutionary history was inferred by using the Maximum Likelihood method and the General Time Reversible model. The percentage of trees in which the associated taxa clustered together is shown next to the branches. Initial tree(s) for the heuristic search were obtained automatically by applying Neighbor-Join and BioNJ algorithms to matrix of pairwise distances estimated using the Maximum Composite Likelihood (MCL) approach and then selecting the topology with superior log likelihood value. The tree is drawn to scale, with branch lengths measured in the number of substitutions per site. This analysis involved 36 nucleotide sequences. There were a total of 1714 positions in the final dataset. Evolutionary analyses were conducted in MEGA12.

**Figure 4:**
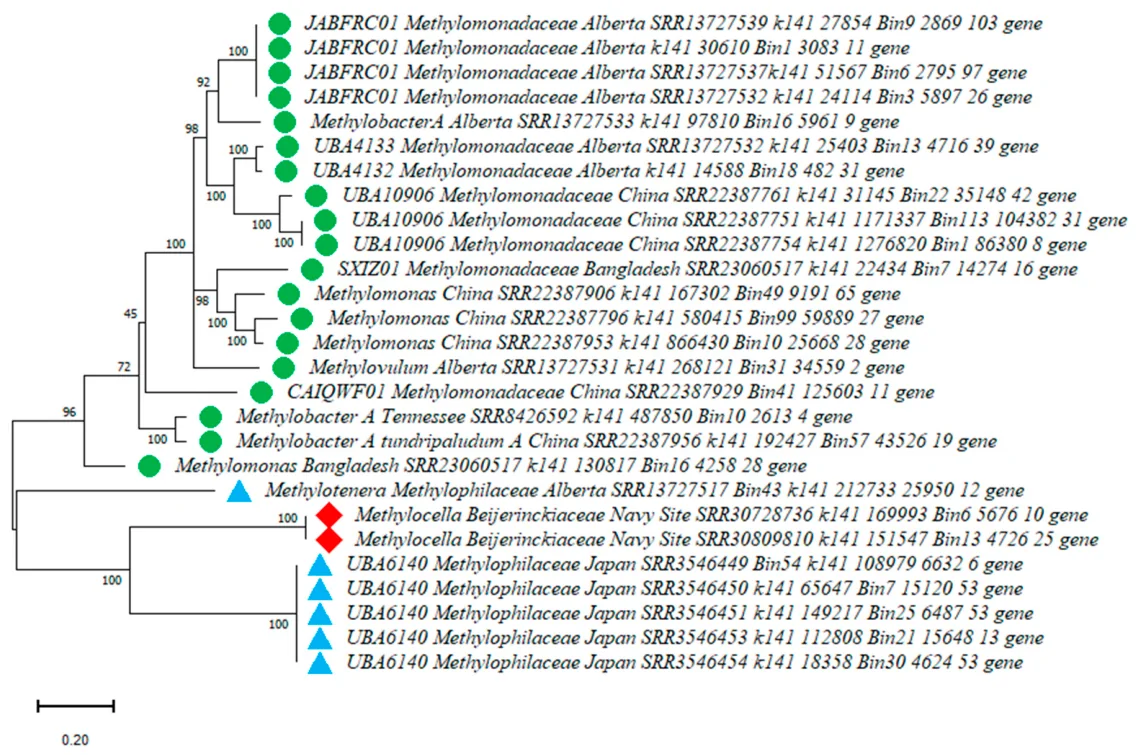
Alignment of *mmoX* from MAGs containing the soluble monooxygenase operon within the *Methylomonadaceae* (green circles), *Methylophilaceae* (blue triangles) and *Beijerinckiaceae* (red diamonds) families. The evolutionary history was inferred by using the Maximum Likelihood method and the General Time Reversible model. The percentage of trees in which the associated taxa clustered together is shown next to the branches. Initial tree(s) for the heuristic search were obtained automatically by applying Neighbor-Join and BioNJ algorithms to a matrix of pairwise distances estimated using the Maximum Composite Likelihood (MCL) approach and then selecting the topology with superior log likelihood value. The tree is drawn to scale, with branch lengths measured in the number of substitutions per site. This analysis involved 27 nucleotide sequences. There were a total of 1605 positions in the final dataset. Evolutionary analyses were conducted in MEGA12.

**Figure 5:**
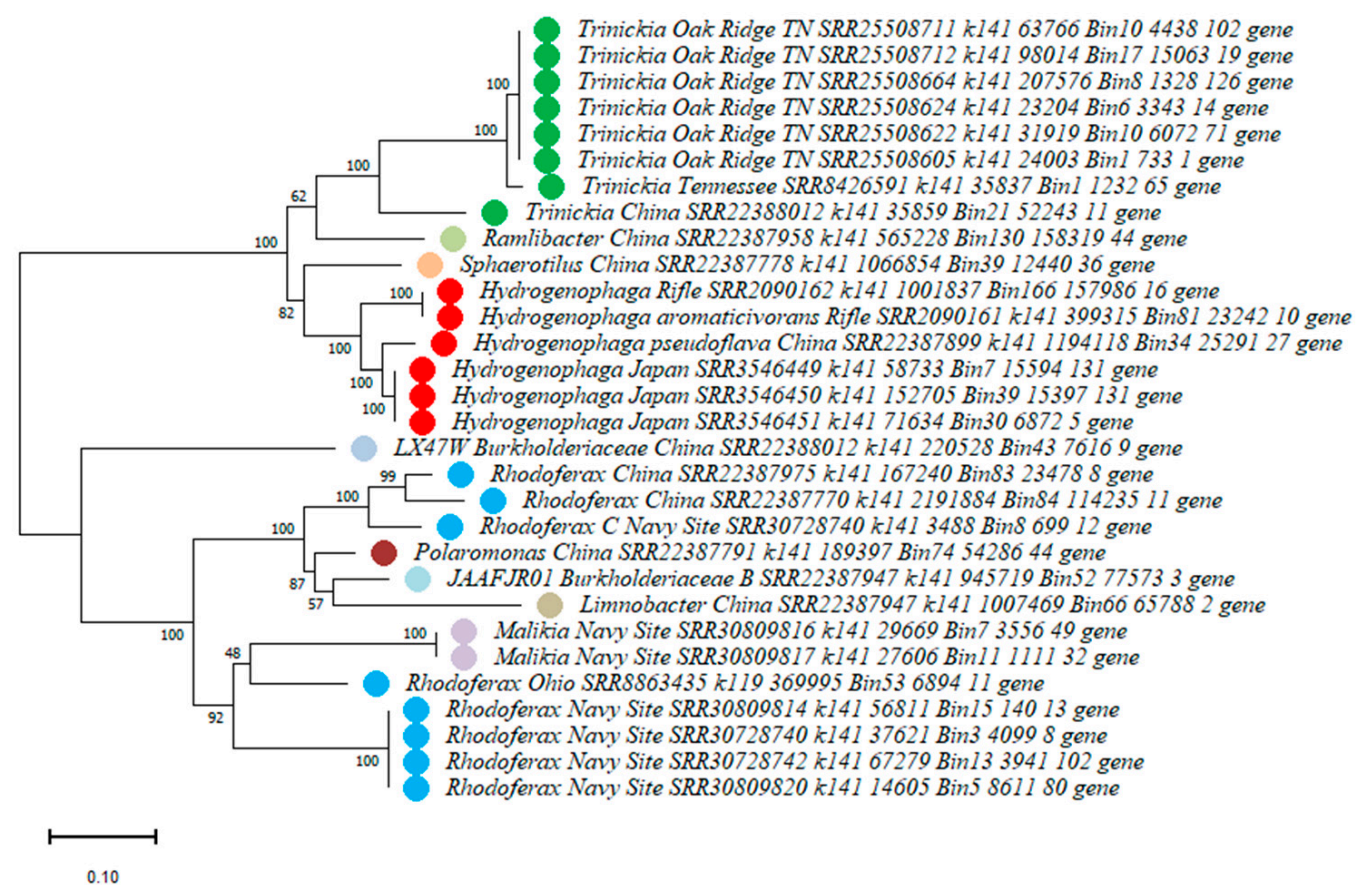
Alignment of *tmoA* from MAGs containing the soluble monooxygenase operon within the family *Burkholderiaceae* (*Burkholderiales*, *Gammaproteobacteria*): *Hydrogenophaga* (red circles), *Rhodoferax* (blue circles), *Malikia* (purple circles), *Sphaerotilus* (orange circle), *Polaromonas* (brown circle), *JAAFJR01* (light blue circle), *Ramlibacter* (light green circle), *Trinickia* (green circle), *Limnobacter* (light brown circle) and *LX47W* (light purple circle). The evolutionary history was inferred by using the Maximum Likelihood method and the General Time Reversible model. The percentage of trees in which the associated taxa clustered together is shown next to the branches. Initial tree(s) for the heuristic search were obtained automatically by applying Neighbor-Join and BioNJ algorithms to a matrix of pairwise distances estimated using the Maximum Composite Likelihood (MCL) approach and then selecting the topology with superior log likelihood value. The tree is drawn to scale, with branch lengths measured in the number of substitutions per site. This analysis involved 30 nucleotide sequences. There were a total of 1508 positions in the final dataset. Evolutionary analyses were conducted in MEGA12.

**Figure 6:**
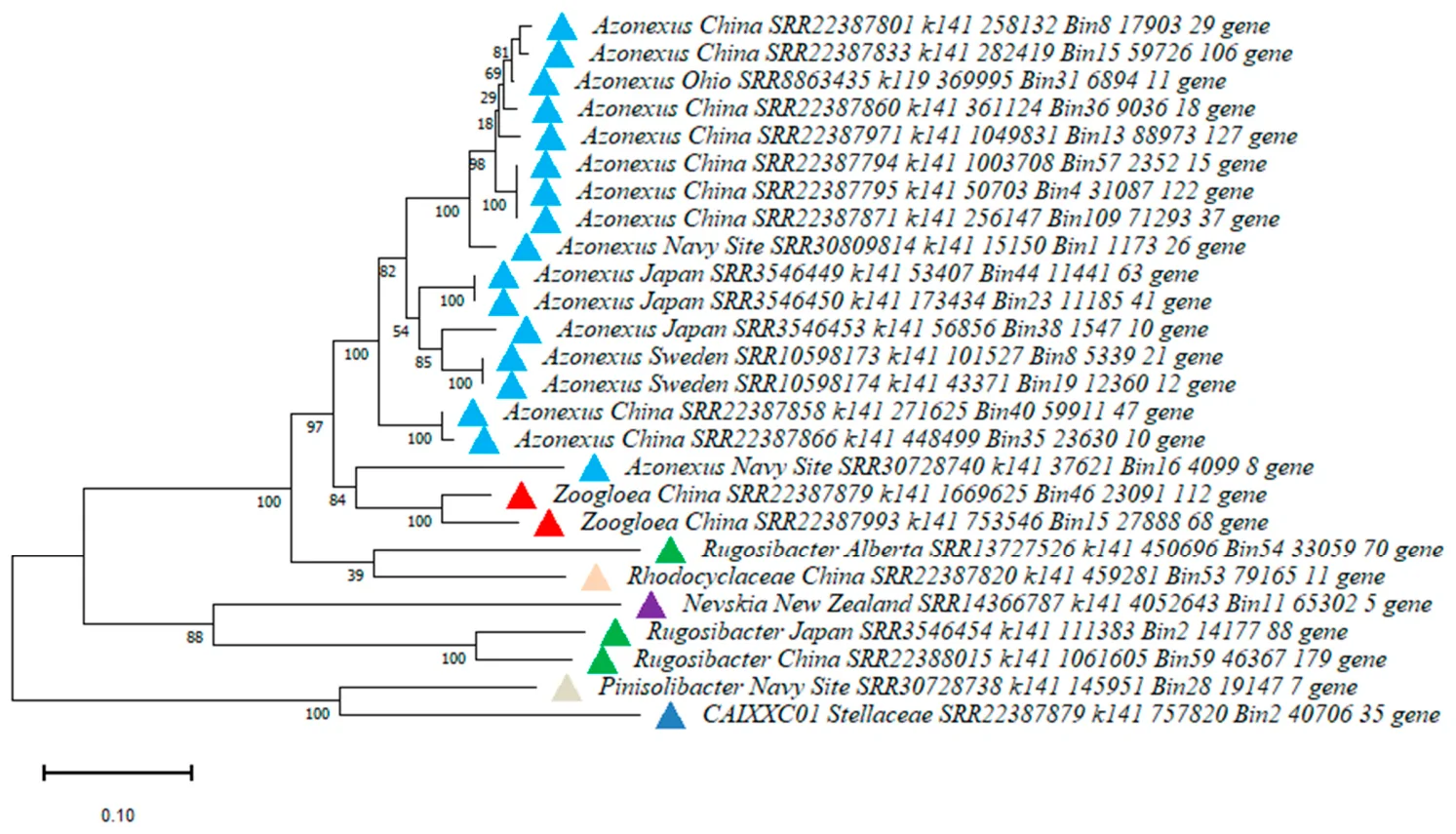
Alignment of *tmoA* from MAGs containing the soluble monooxygenase operon within the *Rhodocyclaceae, Nevskiaceae* (*Gammaproteobacteria*) and *Alphaproteobacteria: Azonexus* (blue triangle), *Rugosibacter* (green triangle), *Zoogloea* (red triangle), *Nevskia* (purple triangle), *Pinisolibacter* (grey triangle), and *CAIXXC01* (dark blue triangle) families. The evolutionary history was inferred by using the Maximum Likelihood method and the General Time Reversible model. The percentage of trees in which the associated taxa clustered together is shown below the branches. Initial tree(s) for the heuristic search were obtained automatically by applying Neighbor-Join and BioNJ algorithms to a matrix of pairwise distances estimated using the Maximum Composite Likelihood (MCL) approach and then selecting the topology with superior log likelihood value. The tree is drawn to scale, with branch lengths measured in the number of substitutions per site. This analysis involved 26 nucleotide sequences. There were a total of 1510 positions in the final dataset. Evolutionary analyses were conducted in MEGA11.

**Figure 7:**
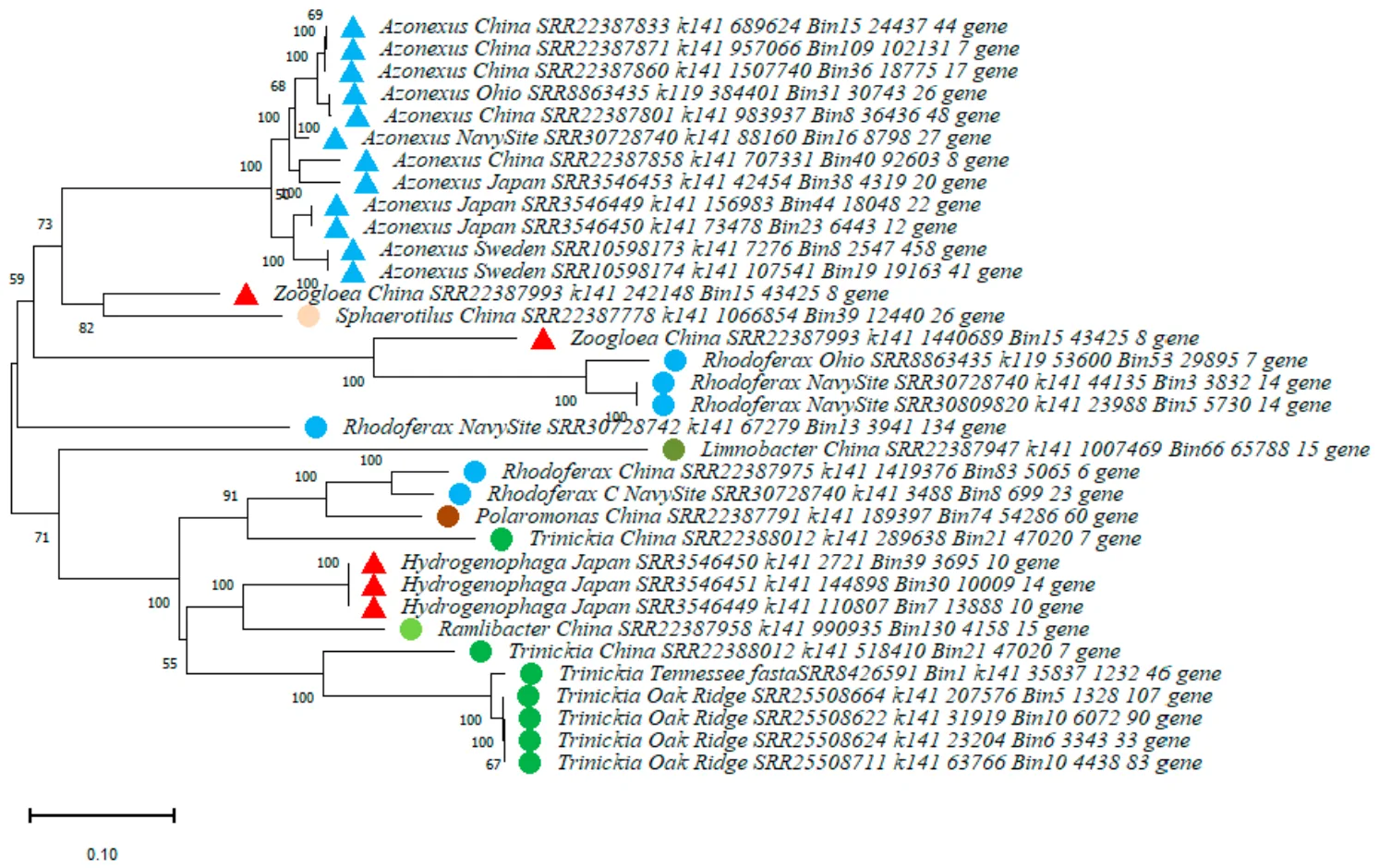
Alignment of *dmpN* from MAGs containing the phenol monooxygenase operon, including *Hydrogenophaga* (red triangle), *Rhodoferax* (blue circles), *Sphaerotilus* (orange circle), *Polaromonas* (brown circle), *Ramlibacter* (light green circle), *Trinickia* (green circle), *Limnobacter* (light brown circle), *Azonexus* (blue triangle), and *Zoogloea* (red triangle). The evolutionary history was inferred by using the Maximum Likelihood method and the General Time Reversible model. The percentage of trees in which the associated taxa clustered together is shown next to the branches. Initial tree(s) for the heuristic search were obtained automatically by applying Neighbor-Join and BioNJ algorithms to a matrix of pairwise distances estimated using the Maximum Composite Likelihood (MCL) approach and then selecting the topology with superior log likelihood value. The tree is drawn to scale, with branch lengths measured in the number of substitutions per site. This analysis involved 34 nucleotide sequences. There were a total of 1573 positions in the final dataset. Evolutionary analyses were conducted in MEGA11.

Phylogenetic trees were constructed for the most common biomarker subunit genes for each operon (Figure 3, Figure 4, Figure 5, Figure 6 and Figure 7 and S11–S13). For propane monooxygenase, *prmA* sequences from each MAG were used to construct the tree (Figure 3) using sequences from the phyla *Actinomycetota*, *Pseudomonadota* and *Chloroflexota* (Supplementary Tables S1 and S2). As expected, *prmA* sequences from the same phyla and families clustered together. For soluble methane monooxygenase, the tree was constructed from *mmoX* sequences from MAGs classifying within *Methylomonadaceae*, *Methylophilaceae*, and *Beijerinckiaceae* (Figure 4). Again, *mmoX* sequences from the same families clustered together. Due to the large number of particulate ammonia/methane monooxygenase operons retrieved, three trees were constructed for *pmoA* (Supplementary Figures S11–S13), and each tree corresponds to data in Supplementary Tables S4, S5 and S6, respectively. For all three trees, *pmoA* sequences from similar phylotypes (order, families, genera) cluster together.

The MAG-derived *tmoA* sequenced were separated into two trees. One tree corresponds to data from MAGs classifying as *Burkholderiaceae* (*Burkholderiales*, *Gammaproteobacteria*) (Supplementary Table S7) (Figure 5). The other tree contains data corresponding to *tmoA* sequences from MAGs classifying within *Rhodocyclaceae*, *Nevskiaceae* (*Gammaproteobacteria*) and *Alphaproteobacteria* (Supplementary Table S8) (Figure 6). For both trees, *tmoA* sequences from similar genera cluster together, e.g., those from *Hydrogenophaga*, *Rhodoferax*, *Trinickia* and *Azonexus*. All *dmpN* sequences obtained from the toluene-containing MAGs are presented in one tree (Figure 7). Similar to the trends illustrated for *tmoA*, *dmpN* sequences from the same genera clustered together.

### 3.3. Di-Iron Center Amino Acid Sequences

The amino acid sequences of both di-iron centers were determined from the *prmA*, *mmoX*, *tmoA* and *dmpN* nucleic acid sequences for all MAGs (Table 2). All MAGs classifying in the phyla *Actinomycetota* and *Chloroflexota* illustrated similar patterns for both di-iron centers for *prmA* (DE**V**RH, DE**S**RH). In contrast, MAGs classifying within the *Pseudomonadota* contained a different amino acid in the first *prmA* di-iron center (DE**F**RH). For *mmoX*, all MAGs contained the same two di-iron center amino acids (DE**I**RH, DE**L**RH). More variability was observed for the first di-iron center of *tmoA*, with four different trends being noted. All second di-iron centers for *tmoA* were the same (DE**S**RH). For *dmpN*, two patterns were present for both di-iron centers (DE**I**RH, DE**L**RH for the first and DE**A**RH and DE**S**RH for the second).

### 3.4. Publicly Available KBase Narratives

As stated in the Methods Section, the MAGs containing the target genes were downloaded from individual narratives, then uploaded to six publicly available KBase narratives. Links to the six KBase narratives can be easily accessed at the following DOI site https://doi.org/10.25982/242870.6/3013398. These narratives are particularly useful, as they provide a user-friendly approach for obtaining nucleotide and amino acid sequence data for the targeted operons and surrounding genes, as well as the size of the genome. Each MAG, with identifying information in Tables S1–S10, is opened in the main window. To retrieve information from any MAG, simply select the browse features tab, then search for the appropriate gene name (“propane”, “methane”, “toluene”, “phenol”, depending on the narrative). This will produce information concerning the start position, strand (+ or −), length and the appropriate contig for each subunit. Following this, select a feature ID (e.g., gene_4358) to get access to the nucleotide and amino acid sequence of each subunit for each operon. Also, in the feature context box generated, users can obtain annotations and sequence data for neighboring genes.

## 4. Discussion

This research involved the analysis of a large amount of whole genome sequencing data (>600 individual samples, >12,000 Gbases, >80 KBase narratives) from groundwater samples to generate groups of MAGs containing the target operons. Following this, the MAGs were uploaded into publicly available KBase narratives to allow others to obtain nucleic and amino acid sequences for all subunits for each operon, as well as the surrounding genes. The number of MAGs identified differed considerably for each target operon: 34 contained the full propane monooxygenase operon; 26 contained the full operon for soluble methane monooxygenase; 55 contained the full operon for toluene monooxygenase; 33 contained both toluene and phenol monooxygenase, and more than 100 contained the full operon for particulate ammonia/particulate methane monooxygenase. The low number of MAGs obtained with the operons of interest is perhaps surprising, given the large amount of data examined. MAGs with toluene monooxygenase and particulate ammonia/methane monooxygenase were detected across 10 and 9 datasets, respectively. In contrast, MAGs with propane monooxygenase and soluble methane monooxygenase were only detected across five and six datasets, respectively. This pattern perhaps indicates the more common occurrence of the former target operons compared to the latter in groundwater. However, additional research is needed to confirm this.

The identification of propane monooxygenase containing MAGs in the phylum *Actinomycetota* is consistent with the results of previous research. Specifically, reported propanotrophs in this phylum include those in the genera *Mycobacterium*, *Rhodococcus*, *Gordonia*, *Nocardioides* and *Pseudonocardia* [2,3,4,90,91,92,93,94,95,96,97]. The current study also identified propane monooxygenase-containing MAGs in the phylum *Pseudomonadota*. Although, based on sequence data [98,99], phylotypes in this phylum have been reported to contain the propane monooxygenase, little is known about their ability to degrade propane. Exceptions to this include *Methylocella* sp. PC1 and PC4, *Methylocella silvestris* BL2, and *Methylocella silvestris* TVC (*Rhizobiales*, *Beijerinckiaceae*), as these can use propane to support growth and contain the propane monooxygenase operon [100,101,102,103]. Also, *Azoarcus* DD4 (*Rhodocyclales*, *Zoogloeaceae)* contains a putative propane monooxygenase and can use propane as a carbon source [104]. Consistent with previous pure and mixed culture work, in the current study for the phylum *Pseudomonadota*, propane monooxygenase-containing MAGs were identified in groundwater from the genera *Methylocella*, *Azoarcus* and *Methylibium*. MAGs in both phyla *Actinomycetota* (*Mycobacterium*, *Rhodococcus opacus*, *Rhodococcus wratislaviensis*, *Pseudonocardia*) and *Pseudomonadota* (*Methylibium*) were also recently identified with the full propane monooxygenase operon in propanotrophic enrichment cultures [9,105].

Methanotrophs classify within two phyla, *Proteobacteria* (classes *Alphaproteobacteria* and *Gammaproteobacteria*) and *Verrucomicrobia* [106]. The genes for sMMO have been well studied in *Methylosinus trichosporium* OB3b [107,108] and *Methylococcus capsulatus* Bath [109,110,111]. In the current study, all MAGs containing sMMO classified within the *Proteobacteria.* Specifically, the majority of sMMO operons were detected in MAGs in the family *Methylomonadaceae* (*Methylococcales*, *Gammaproteobacteria*), including, for example, those in the genera *Methylobacter*, *Methylovulum* and *Methylomonas.* Consistent with this, sMMO genes have previously been reported for species in the same genera: *Methylobacter methanoversatilis*, *Methylobacter tundripaludum*, *Methylobacter psychrophilus* [112], *Methylovulum miyakonense* HT12 [113], *Methylomonas* sp. strain 11b, *Methylomonas* sp. strain LW13 [114], *Methylomonas methanica* strain 68-1 [115], and *Methylomonas methanica* MC09 [116]. Two *Methylocella* (*Beijerinckiaceae*, *Rhizobiales*, *Alphaproteobacteria*) MAGs containing sMMO operons were also identified in the current study. This is also consistent with previous reports of this genus containing sMMO, for example, for three strains of *Methylocella palustris* [117], *Methylocella silvestris* BL2^T^ [118], and three strains of *Methylocella tundrae* [119]. The identification of sMMO-containing MAGs in the family *Methylophilaceae* is more puzzling, as this family typically utilizes methanol or methylamine as a sole source of carbon and energy [120,121]. As the MAG was only classified to the family level, it is possible that a mis-identification occurred. It is surprising that no MAGs containing sMMO were detected in the genera *Methylosinus*, *Methylococcus Methylocystis*, as these phylotypes have previously been detected in groundwater aquifers [122,123].

The majority of MAGs containing the full operon for ammonia/particulate methane monooxygenase classified within the *Gammaproteobacteria* (*Methylococcales*, *Burkholderiales*, *Pseudomonadales* and *Nevskiales*), with only six within the *Alphaproteobacteria* (primarily *Rhizobiales*). The remaining MAGs with this operon classified with the phyla *Methylomirabilota*, *Bacteroidota*, *CSP1-3*, *Nitrospirota*, *Actinomycetota* and *Desulfobacterota_B*. Others have also documented the presence of *Alphaproteobacteria* and *Gammaproteobacteria* containing this operon in groundwater. For example, *Methylobacter* spp. were active in groundwater microcosms capable of the biodegradation of chlorinated volatile organic compounds [124]. In another study focused on the stimulation of methanotrophs in multiple acidic aquifers, various genera (*Methylomonas*, *Methylobacter*, *Methylosinus*, and *Methylococcus*) were detected [122]. Molecular analysis of uncontaminated groundwater wells and methanotrophic enrichment cultures derived from the water also revealed sequences aligning with the genera *Methylobacter*, *Methylosinus*, *Methylomonas*, *Methylocaldum*, and *Methylocystis* [123]. Little is known about the occurrence of methanotrophs with this operon in the additional phyla listed above.

MAGs containing the full operon for toluene monooxygenase classified within the *Burkholderiaceae* (*Burkholderiales*, *Gammaproteobacteria*), *Rhodocyclaceae* (*Burkholderiales*, *Gammaproteobacteria*) and the *Alphaproteobacteria* families. The majority classified in a small number of genera, including *Azonexus*, *Hydrogenophaga*, *Rhodoferax* and *Trinickia*. The detection of the genes encoding for toluene monooxygenase has significant implications, as this enzymes has been linked to the biodegradation of numerous pollutants, such as alkenes, 1,4-dioxane, N-nitrosodimethylamine, chloroform, TCE, various polycyclic aromatic substrates, and substituted benzenes [24,25,26,27,28,29,30,31]. For example, *tmoABCDEF* has been associated with the co-metabolism of 1,1-DCE, *cis*-DCE, and 1,4-dioxane in *Azoarcus* sp. DD4 [22,23]. The same gene cluster was found within a *Rhodoferax* MAG in enrichment cultures capable of xylene degradation, established from groundwater from a xylene-contaminated site [70]. Additional research will be needed to more conclusively link contaminant biodegradation to each toluene monooxygenase-containing MAG.

## 5. Conclusions

The current study provides novel gene sequences and MAGs from groundwater metagenomes for a group of important biomarkers for the aerobic biodegradation of pollutants. Of course, it is important to state that the presence of genetic material does not necessarily indicate biodegradation activity. Such results have important implications because these functional genes have been associated with the removal of numerous groundwater contaminants. The work highlights the advantages of KBase for the analysis of large quantities of whole genome sequencing data. Further, KBase narratives are publicly available, providing an excellent platform for sharing data. For example, qPCR assays could be updated based on the collected sequences. From the biomarkers investigated, MAGs containing particulate methane/ammonia monooxygenase were most commonly detected (>100). Multiple (>20 for each) MAGs containing the full operons for propane monooxygenase, soluble methane monooxygenase, toluene monooxygenase and phenol monooxygenase were also detected. The MAGs generated and their associated functional gene sequences have the potential to improve molecular detection methods for site bioremediation.

## Figures and Tables

**Table 1:** Examined whole genome sequencing datasets obtained from NCBI’s SRA.

BioProject Accession	Site Information	Number of Samples and the Total Number of Bases Analyzed in the Current Study	Reference
PRJNA288027	Groundwater metagenomes, Rifle, CO, USA	25 samples, ~500 Gbases	[37,38,39]
PRJNA512237	Groundwater metagenomes, Ohio	9 samples, ~13 Gbases	[40]
PRJNA699054	Groundwater and sediment metagenomes, New Zealand	25 samples, ~55 Gbases	[41]
PRJNA513876	Groundwater metagenomes, Tennessee, USA	12 samples, ~400 Gbases	[42]
PRJNA1001011	Sediment and groundwater metagenomes from subsurface microbial communities from the Oak Ridge National Laboratory Field Research Center, Oak Ridge, TN, USA	114 samples, ~926 Gbases	[43,44]
PRJNA321556	Groundwater metagenomes, Horonobe Underground Research Laboratory, Hokkaido, Japan	9 samples, ~130 Gbases	[45]
PRJNA700657	Groundwater metagenomes, Alberta, Cananda	26 samples, ~395 Gbases	[46]
PRJNA1162924	US Navy Site, USA	22 samples, ~107 Gbases	[47]
PRJNA279923	Metagenome of deep subsurface groundwater in Äspö, Sweden	15 samples, ~120 Gbases	[48]
PRJNA858913	Groundwater metagenomes from samples from monitoring wells in seven geo-environment zones across China	>300 samples, >30 Gbases per sample	[49]
PRJNA916093	Groundwater metagenomes, Bangladesh	6 samples, ~22 Gbases	[50]
PRJEB36505	Groundwater metagenomes, within the Hainich Critical Zone Exploratory, Turingia, Germany	32 samples, ~626 Gbases	[51]
PRJNA947390	Groundwater metagenomes from three groundwater monitoring wells in an agricultural area in the Netherlands	18 samples, ~112 Gbases	[52]

**Table 2:** Amino acid composition of both di-iron centers (DE*RH) in the MAGs.

Operon and MAG	First Di-Iron Center	Second Di-Iron Center
**Propane monooxygenase**		
All MAGs in the phyla *Actinomycetota and Chloroflexota*	DE **V** RH	DE **S** RH
All MAGs in the phylum *Pseudomonadota*	DE **F** RH	DE **S** RH
**Soluble methane monooxygenase**		
All MAGs	DE **I** RH	DE **L** RH
**Toluene monooxygenase**		
***Rhodocyclaceae, Nevskiaceae (Gammaproteobacteria), Alphaproteobacteria***		
*Azonexus (all except 1)*	DE **I** RH	DE **S** RH
*Rugosibacter (2 of 3)*	DE **I** RH	DE **S** RH
*Zoogloea*	DE **I** RH	DE **S** RH
*Nevskia*	DE **I** RH	DE **S** RH
*Azonexus (1)*	DE **V** RH	DE **S** RH
*Rugosibacter (1 of 3)*	DE **V** RH	DE **S** RH
*Pinisolibacter*	DR **N** RH	DE **S** RH
***Burkholderiaceae (Burkholderiales, Gammaproteobacteria)***		
*Hydrogenophaga*	DR **N** RH	DE **S** RH
*Sphaerotilus*	DR **N** RH	DE **S** RH
*Ramlibacter*	DR **N** RH	DE **S** RH
*Trinickia*	DR **N** RH	DE **S** RH
*Stellaceae*	DR **N** RH	DE **S** RH
*Rhodoferax (1)*	DE **I** RH	DE **S** RH
*Burkholderiaceae*	DE **I** RH	DE **S** RH
*Limnbacter*	DE **I** RH	DE **S** RH
*LX47W*	DE **I** RH	DE **S** RH
*Polaromonas*	DE **M** RH	DE **S** RH
*Rhodoferax (3)*	DE **M** RH	DE **S** RH
*Malikia*	DE **V** RH	DE **S** RH
*Rhodoferax (4)*	DE **V** RH	DE **S** RH
**Phenol monooxygenase**		
***Rhodocyclaceae, Nevskiaceae (Gammaproteobacteria), Alphaproteobacteria***		
*Azonexus*	DE **I** RH	DE **A** RH
*Zoogloea*	DE **I** RH	DE **A** RH
***Burkholderiaceae (Burkholderiales, Gammaproteobacteria)***		
*Hydrogenophaga*	DE **L** RH	DE **S** RH
*Sphaerotilus*	DE **I** RH	DE **A** RH
*Ramlibacter*	DE **L** RH	DE **S** RH
*Trinickia*	DE **L** RH	DE **S** RH
*Limnobacter*	DE **L** RH	DE **S** RH

## Data Availability

All sequencing data was obtained from NCBI. Links to all KBase narratives containing all MAGs are available using the following narrative: “Data Mining of Groundwater to Identify MAGs with Methane, Propane and Toluene Monooxygenases” (https://narrative.kbase.us/narrative/242870, accessed on 1 July 2025).

## Supplementary Materials

The following supporting information can be downloaded at: https://www.mdpi.com/article/10.3390/biology15181586/s1, Supplementary Figure S1. Subunit length and arrangement of soluble monooxygenase operons in groundwater metagenomes associated with the identified metagenome assembled genomes within the *Gammaproteobacteria* and *Alphaproteobacteria*, Supplementary Figure S2. Subunit length and arrangement of soluble monooxygenase operons in groundwater metagenomes associated with the identified metagenome assembled genomes within the *Gammaproteobacteria* and *Alphaproteobacteria*. The *mmoD* subunit was not annotated in these cases. Supplementary Figure S3. Subunit length and arrangement of ammonia/methane monooxygenase operons in groundwater metagenomes associated with the identified metagenome assembled genomes within the *Methylococcales* (*Gammaproteobacteria*). Supplementary Figure S4. Subunit length and arrangement of ammonia/methane monooxygenase operons in groundwater metagenomes associated with the identified metagenome assembled genomes within the *Burkholderiales* and *Pseudomonadales* and *Nevskiales* (*Gammaproteobacteria*). Supplementary Figure S5. Subunit length and arrangement of ammonia/methane monooxygenase operons in groundwater metagenomes associated with the identified metagenome assembled genomes within the the class *Alphaproteobacteria*, and *phyla Methylomirabilota, Bacteroidota, CSP1-3*, *Nitrospirota*, *Actinomycetota* and *Desulfobacterota*_*B*. Supplementary Figure S6. Subunit length and arrangement of toluene monooxygenase operons in groundwater metagenomes associated with the identified metagenome assembled genomes within the *Burkholderiaceae* (*Burkholderiales*, *Gammaproteobacteria*). Supplementary Figure S7. Subunit length and arrangement of toluene monooxygenase operons in groundwater metagenomes associated with the identified metagenome assembled genomes within the *Rhodocyclaceae*, *Nevskiaceae* (*Gammaproteobacteria*) and *Alphaproteobacteria*. Supplementary Figure S8. Subunit length and arrangement of phenol monooxygenase operons in groundwater metagenomes associated with *Azonexus* metagenome assembled genomes also containing toluene monooxygenase operons. Supplementary Figure S9. Subunit length and arrangement of phenol monooxygenase operons in groundwater metagenomes associated with *Hydrogenophaga*, *Ramlibacter*, *Rhodoferax*, *Trinickia* and *Zoogloea* metagenome assembled genomes also containing toluene monooxygenase operons. Supplementary Figure S10. Subunit length and arrangement of phenol monooxygenase operons in close proximity to a toluene monooxygenase operon. Supplementary Figure S11. Alignment of *pmoA* from MAGs containing the particulate ammonia/methane monooxygenase operon within the *Methylococcales* (*Gammaproteobacteria*). Supplementary Figure S12. Alignment of *pmoA* from MAGs containing the particulate ammonia/methane monooxygenase operon within the *Burkholderiales* and *Pseudomonadales* and *Nevskiales* (*Gammaproteobacteria*). Supplementary Figure S13. Alignment of *pmoA* from MAGs containing the particulate ammonia/methane monooxygenase operon within the class *Alphaproteobacteria*, and phyla *Methylomirabilota*, *Bacteroidota, CSP1-3*, *Nitrospirota*, *Actinomycetota* and *Desulfobacterota_B*. Supplementary Table S1. MAGs containing four subunits of propane monooxygenase classifying within the phylum *Actinomycetota*. Supplementary Table S2. MAGs containing four subunits of propane monooxygenase classifying within the phyla *Pseudomonadota* and *Chloroflexota*. Supplementary Table S3. MAGs containing soluble methane monooxygenase operons classifying within the *Gammaproteobacteria* and *Alphaproteobacteria*. Supplementary Table S4. MAGs containing particulate ammonia/particulate methane monooxygenase operons classifying within the *Methylococcales* (*Gammaproteobacteria*). Supplementary Table S5. MAGs containing particulate ammonia/particulate methane monooxygenase operons classifying within the *Burkholderiales* and *Pseudomonadales* and *Nevskiales* (*Gammaproteobacteria*). Supplementary Table S6. MAGs containing particulate ammonia/particulate methane monooxygenase operons classifying within the class *Alphaproteobacteria*, and phyla *Methylomirabilota*, *Bacteroidota*, *CSP1-3*, *Nitrospirota*, *Actinomycetota* and *Desulfobacterota_B*. Supplementary Table S7. MAGs containing toluene monooxygenase operons classifying within the family *Burkholderiaceae* (*Burkholderiales*, *Gammaproteobacteria*). Supplementary Table S8. MAGs containing toluene monooxygenase operons classifying within the *Rhodocyclaceae*, *Nevskiaceae* (*Gammaproteobacteria*) and *Alphaproteobacteria*. Supplementary Table S9. MAGs containing toluene monooxygenase operons classifying within the family *Burkholderiaceae* (*Burkholderiales*, *Gammaproteobacteria*). Supplementary Table S10. MAGs containing both toluene monooxygenase and phenol monooxygenase operons within the *Rhodocyclaceae*.
