# Supplementary material for: Data Mining of Groundwater Genomes for Metagenome-Assembled Genomes (MAGs) Containing Monooxygenase Operons Associated with Contaminant Biodegradation
Source: Biology (Basel). 2026 Sep 9;15(18):1586. doi: 10.3390/biology15181586 (PMC13603553; doi:10.3390/biology15181586)
Supplement: Supplementary file 1 [file biology-15-01586-s001.zip › biology-4519505-supplementary.pdf]

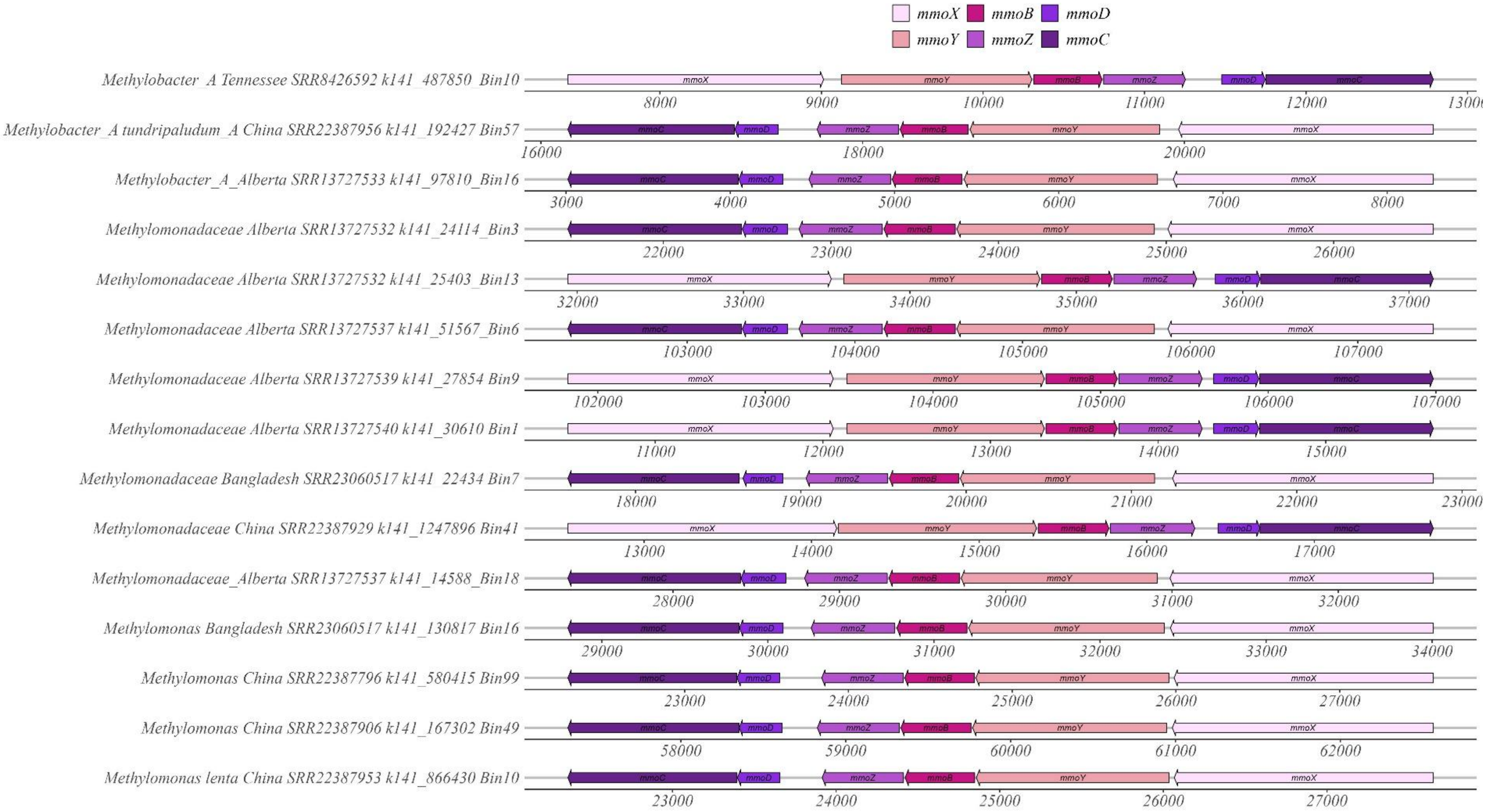

Supplementary Figure S1. Subunit length and arrangement of soluble monooxygenase operons in groundwater metagenomes associated with the identified metagenome assembled genomes within the *Gammaproteobacteria* and *Alphaproteobacteria.*

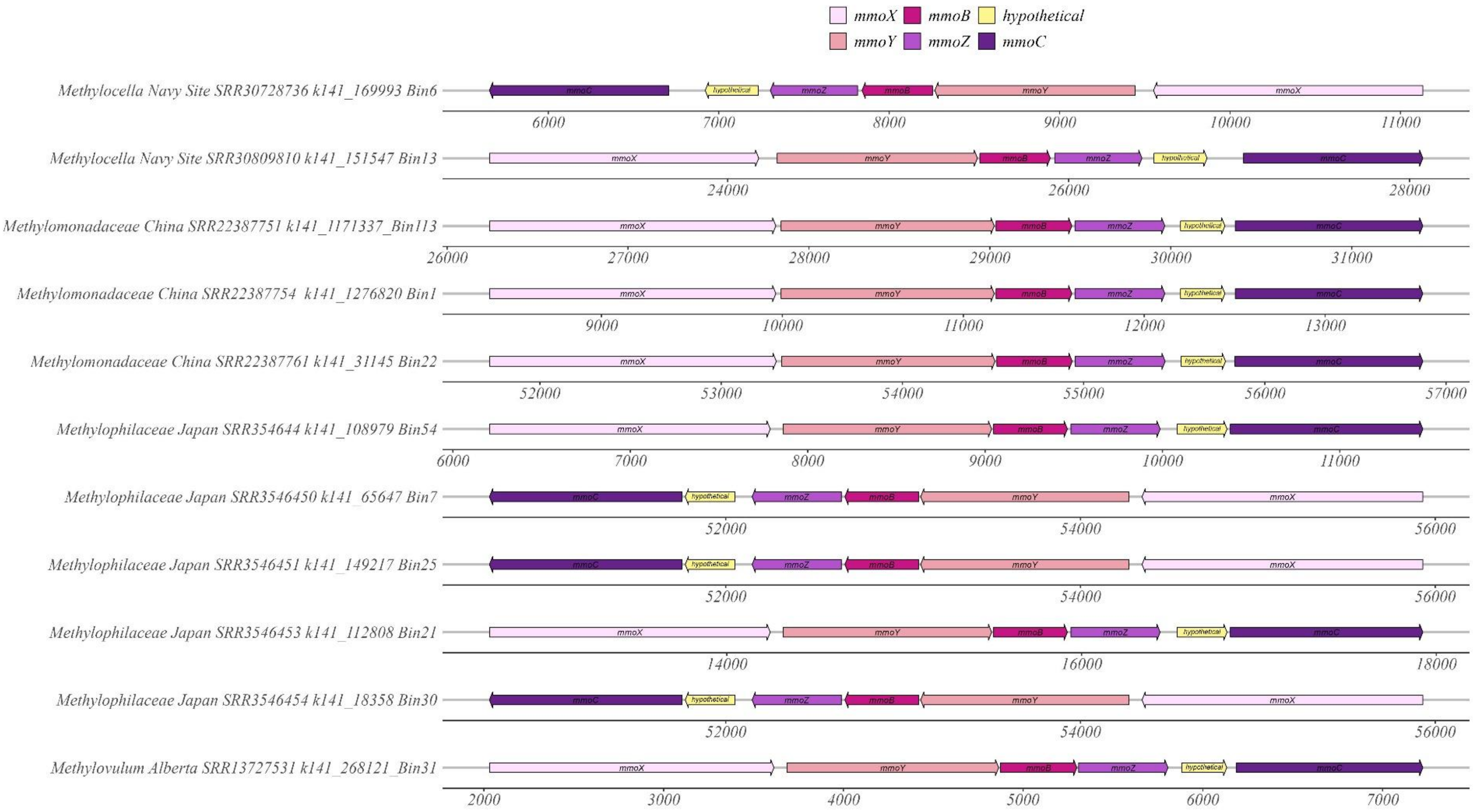

Supplementary Figure S2. Subunit length and arrangement of soluble monooxygenase operons in groundwater metagenomes associated with the identified metagenome assembled genomes within the *Gammaproteobacteria* and *Alphaproteobacteria.* The *mmoD* subunit was not annotated in these cases.

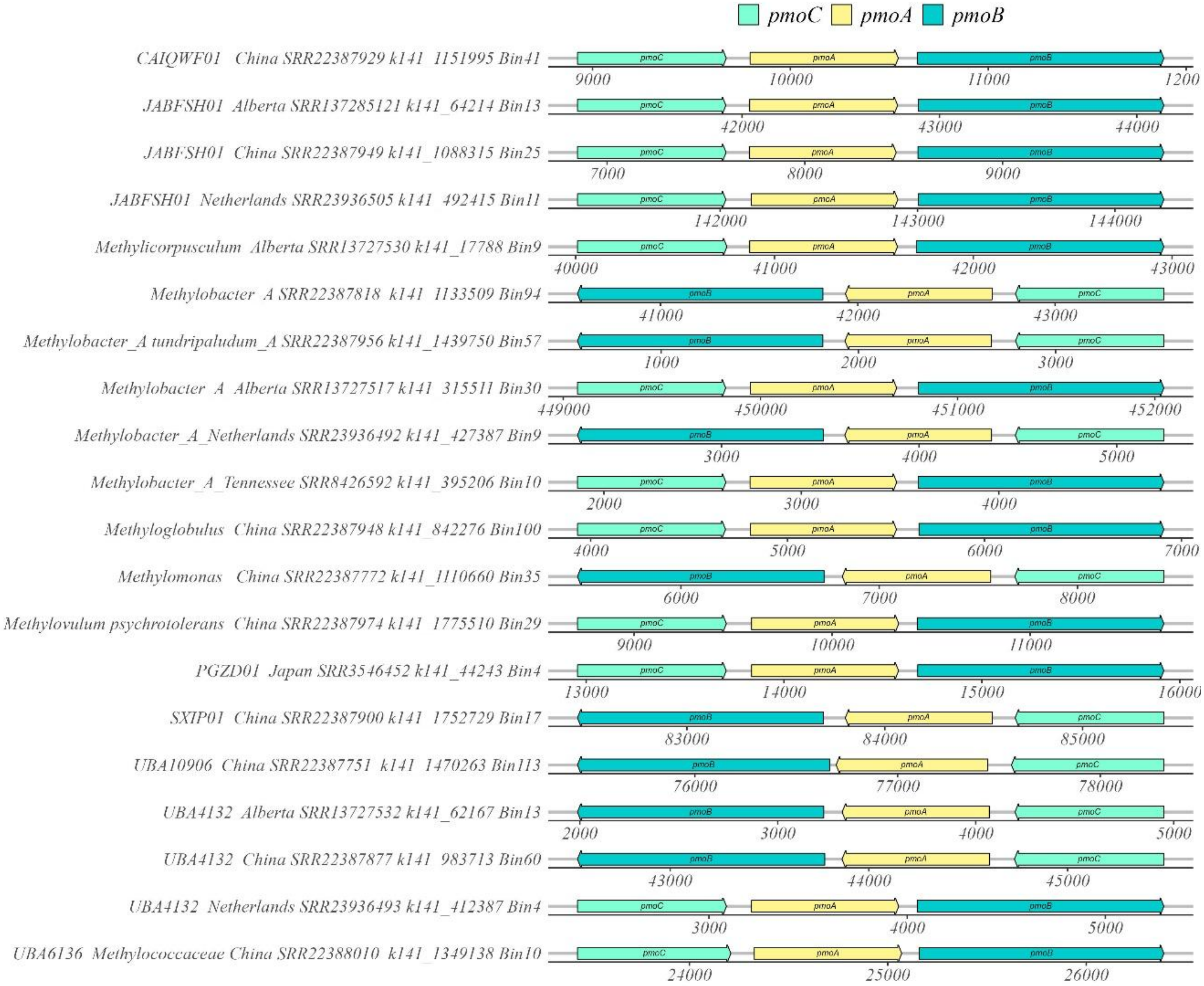

Supplementary Figure S3. Subunit length and arrangement of ammonia/methane monooxygenase operons in groundwater metagenomes associated with the identified metagenome assembled genomes within the *Methylococcales (Gammaproteobacteria)*.

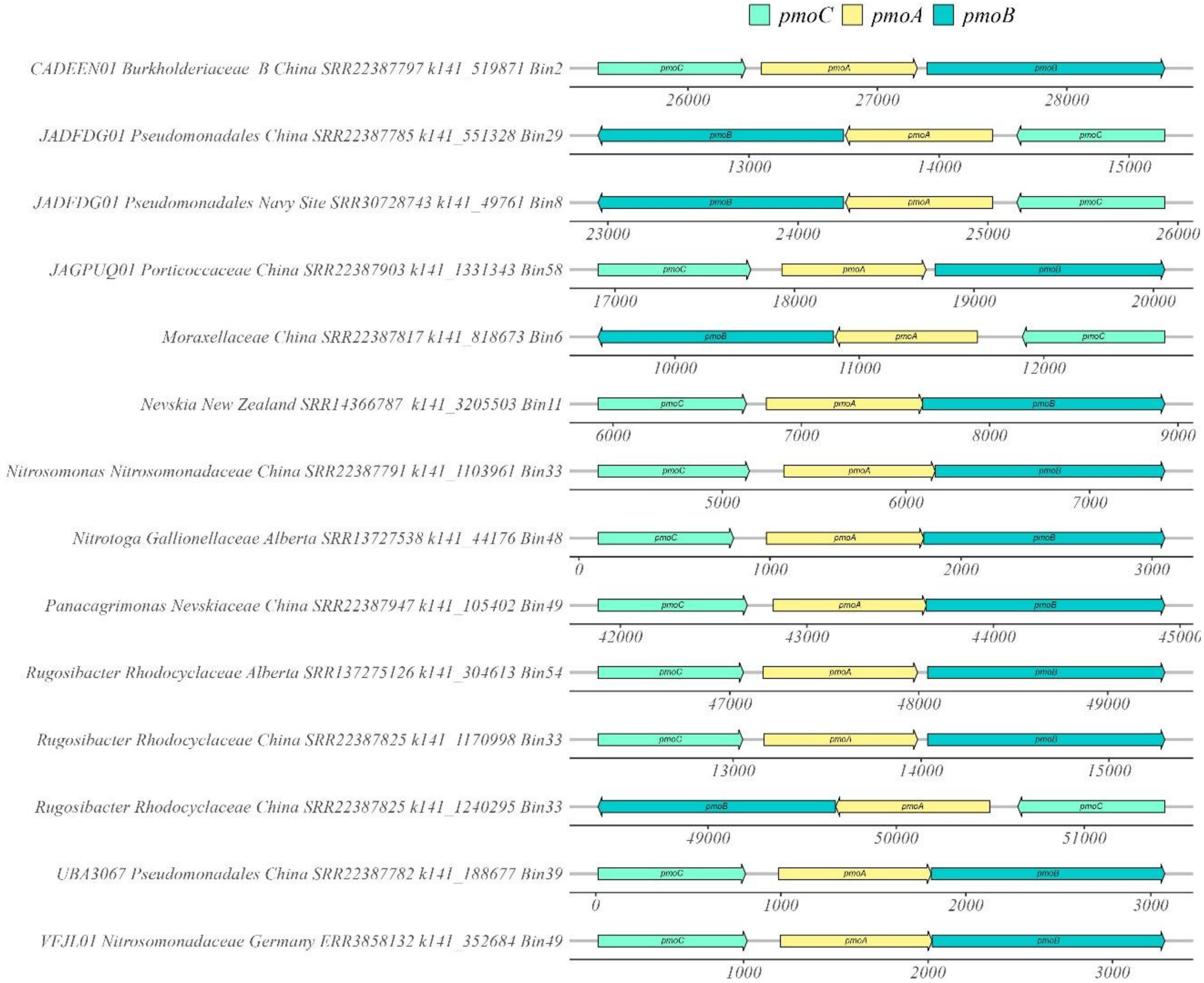

Supplementary Figure S4. Subunit length and arrangement of ammonia/methane monooxygenase operons in groundwater metagenomes associated with the identified metagenome assembled genomes within the *Burkholderiales* and *Pseudomonadales* and *Nevskiales (Gammaproteobacteria)*.

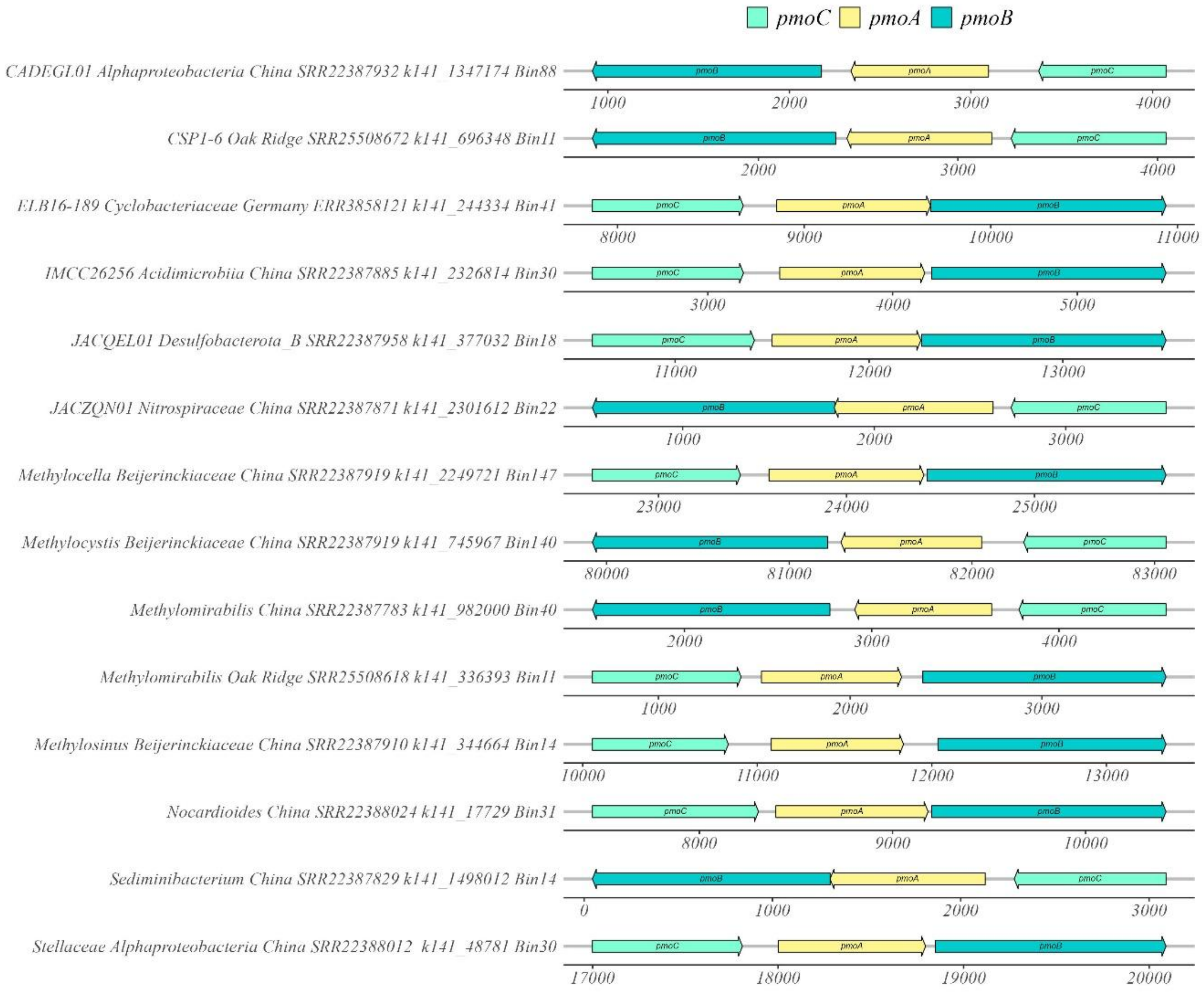

Supplementary Figure S5. Subunit length and arrangement of ammonia/methane monooxygenase operons in groundwater metagenomes associated with the identified metagenome assembled genomes within the the class *Alphaproteobacteria,* and phyla *Methylomirabilota, Bacteroidota, CSP1-3, Nitrospirota, Actinomycetota* and *Desulfobacterota_B.*

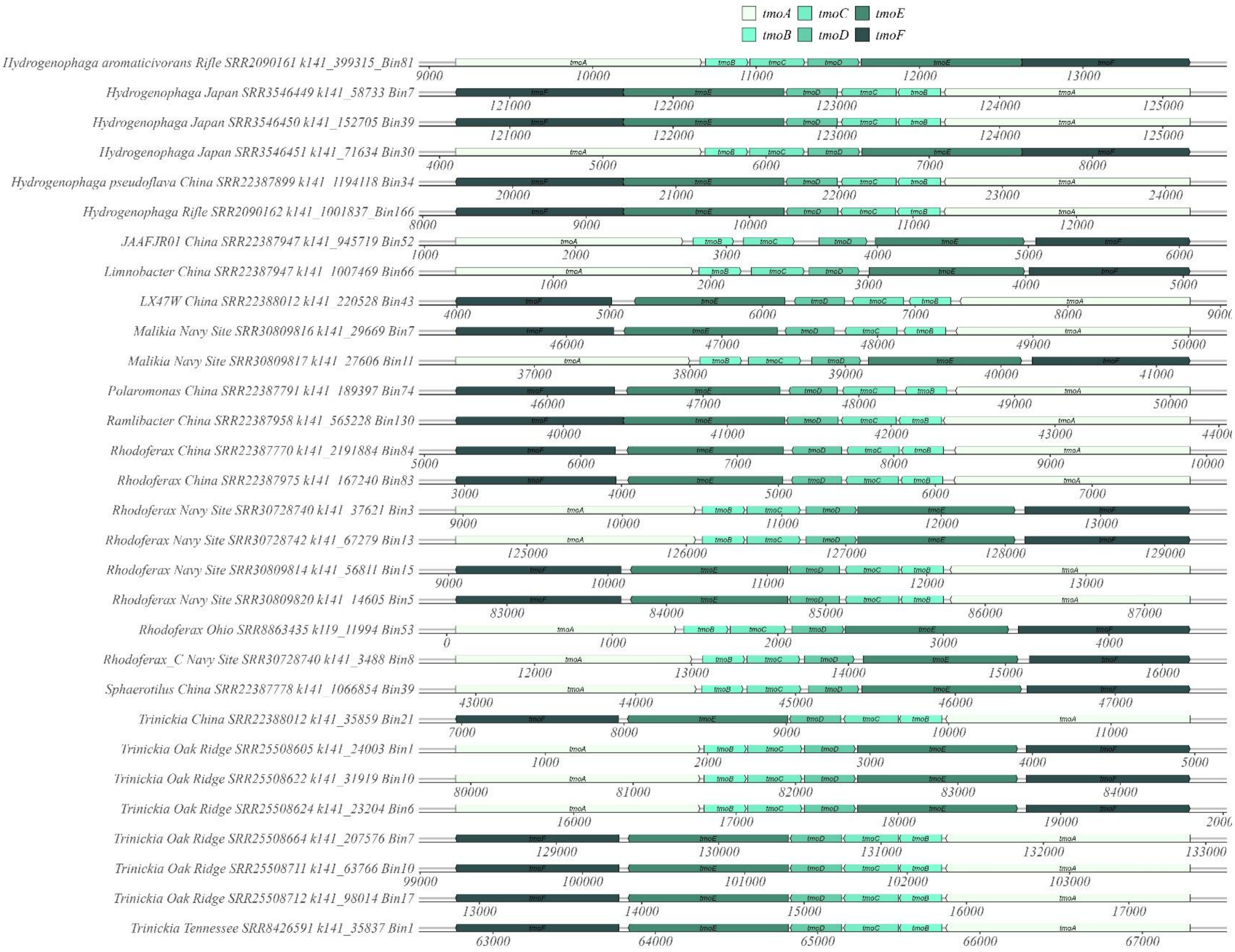

Supplementary Figure S6. Subunit length and arrangement of toluene monooxygenase operons in groundwater metagenomes associated with the identified metagenome assembled genomes within the *Burkholderiaceae (Burkholderiales, Gammaproteobacteria).*

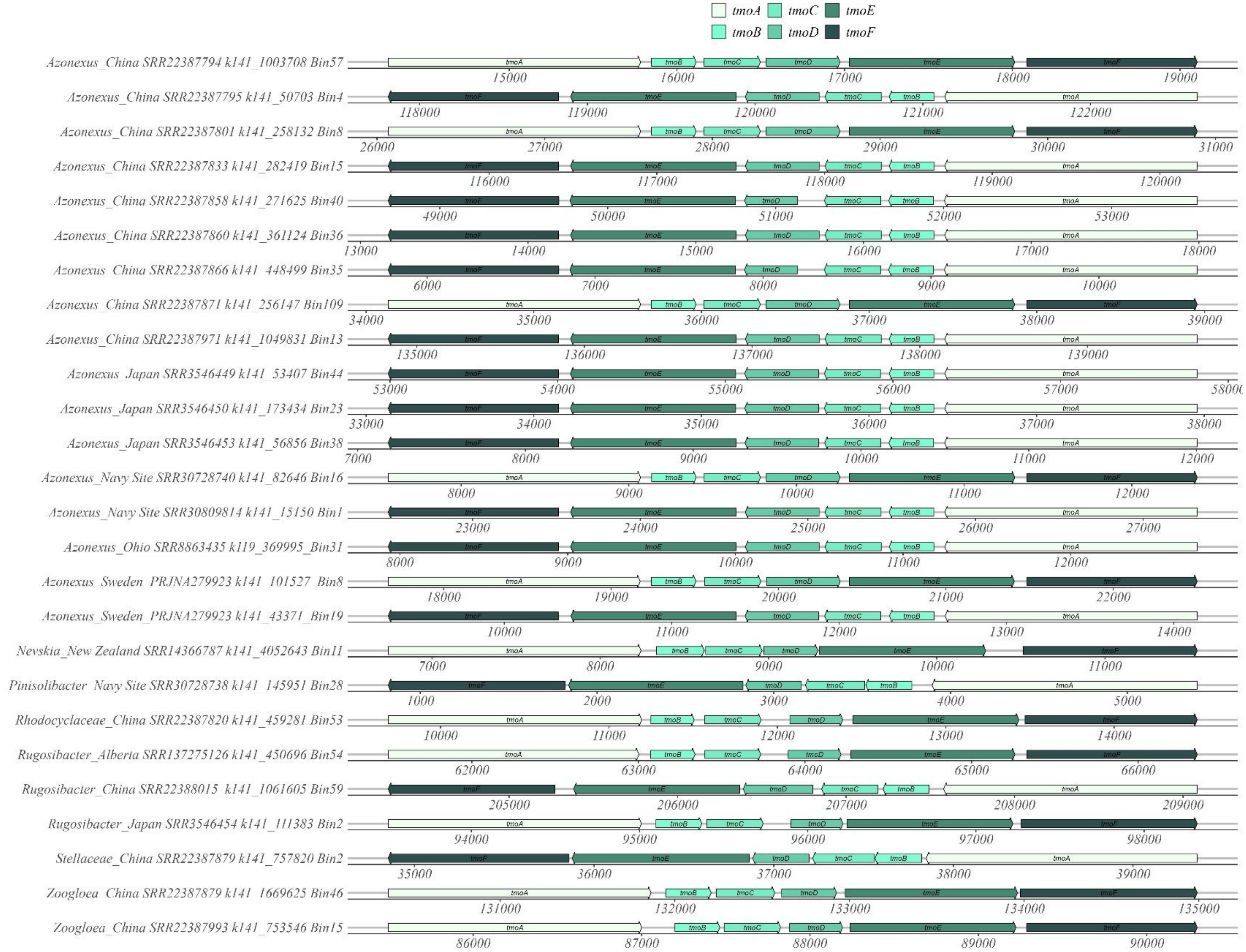

Supplementary Figure S7. Subunit length and arrangement of toluene monooxygenase operons in groundwater metagenomes associated with the identified metagenome assembled genomes within the *Rhodocyclaceae, Nevskiaceae (Gammaproteobacteria)* and *Alphaproteobacteria.*

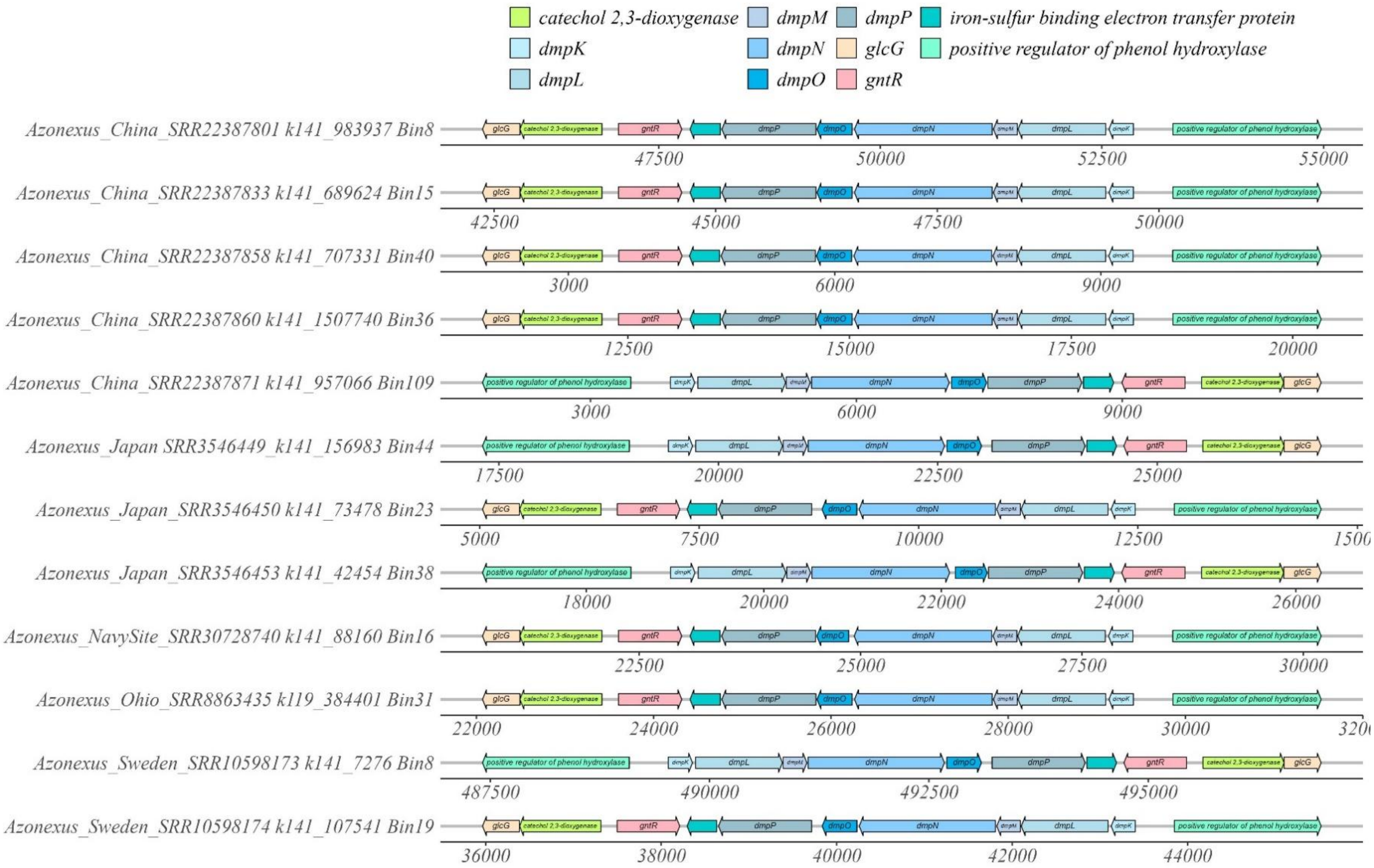

Supplementary Figure S8. Subunit length and arrangement of phenol monooxygenase operons in groundwater metagenomes associated with *Azonexus* metagenome assembled genomes also containing toluene monooxygenase operons. The operons contain catechol 2,3-dioxygenase, phenol hydroxylase assembly protein (*dmpK*), P1 oxygenase component (*dmpL*), P2 regulatory component (*dmpM*), P3 oxygenase component (*dmpN*), P4 oxygenase component (*dmpO*), FAD- and [2Fe-2S]-containing reductase component (*dmpP*), protein GlcG (*glcG*), regulatory protein GntR (*gntR*), irom-sulfur binding electron transfer protein, and positive regulator of phenol hydroxylase.

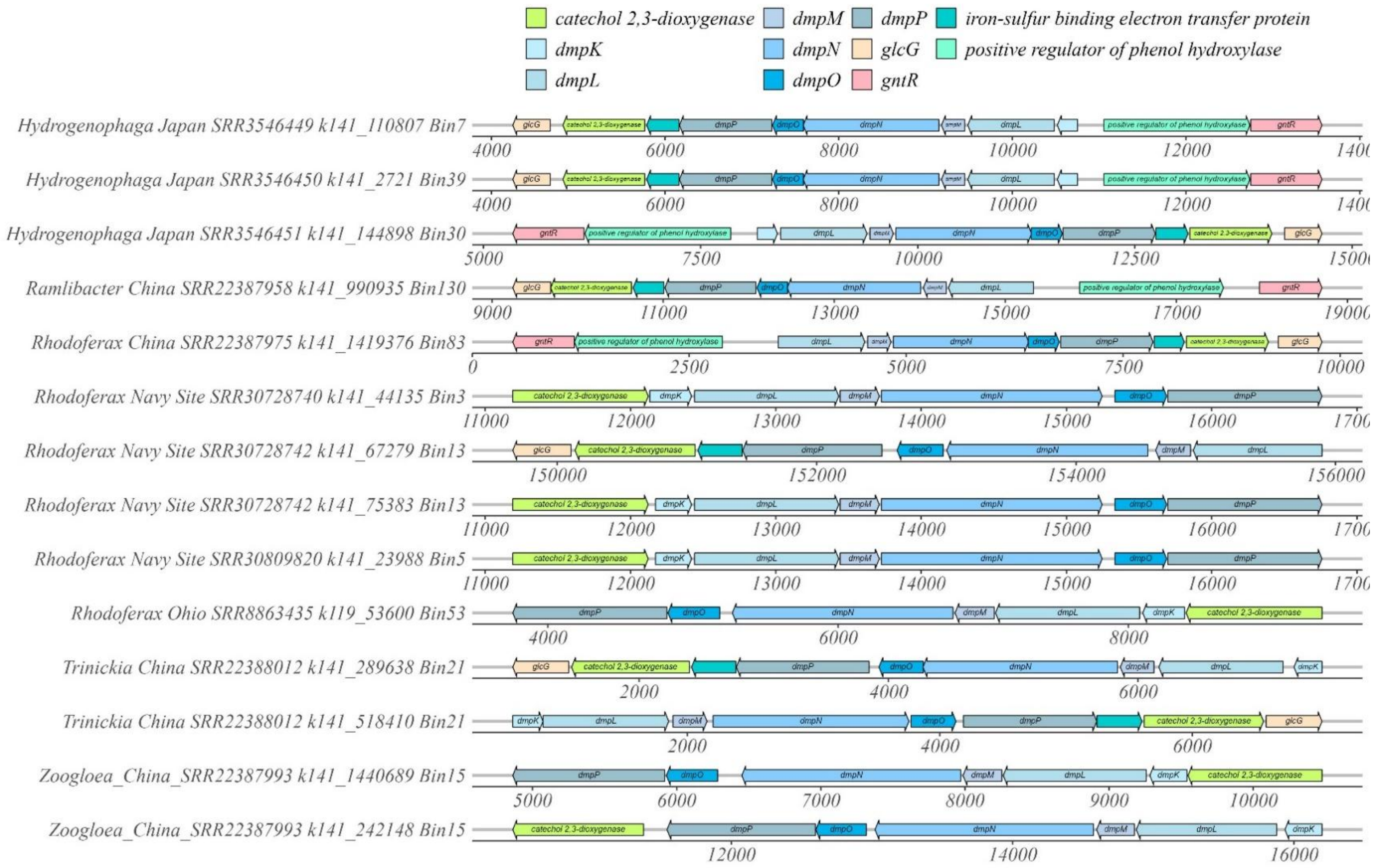

Supplementary Figure S9. Subunit length and arrangement of phenol monooxygenase operons in groundwater metagenomes associated with *Hydrogenophaga, Ramlibacter, Rhodoferax, Trinickia* and *Zoogloea* metagenome assembled genomes also containing toluene monooxygenase operons. The operons contain catechol 2,3-dioxygenase, phenol hydroxylase assembly protein (*dmpK*), P1 oxygenase component (*dmpL*), P2 regulatory component (*dmpM*), P3 oxygenase component (*dmpN*), P4 oxygenase component (*dmpO*), FAD- and [2Fe-2S]-containing reductase component (*dmpP*), protein GlcG (*glcG*), regulatory protein GntR (*gntR*), irom-sulfur binding electron transfer protein, and positive regulator of phenol hydroxylase.

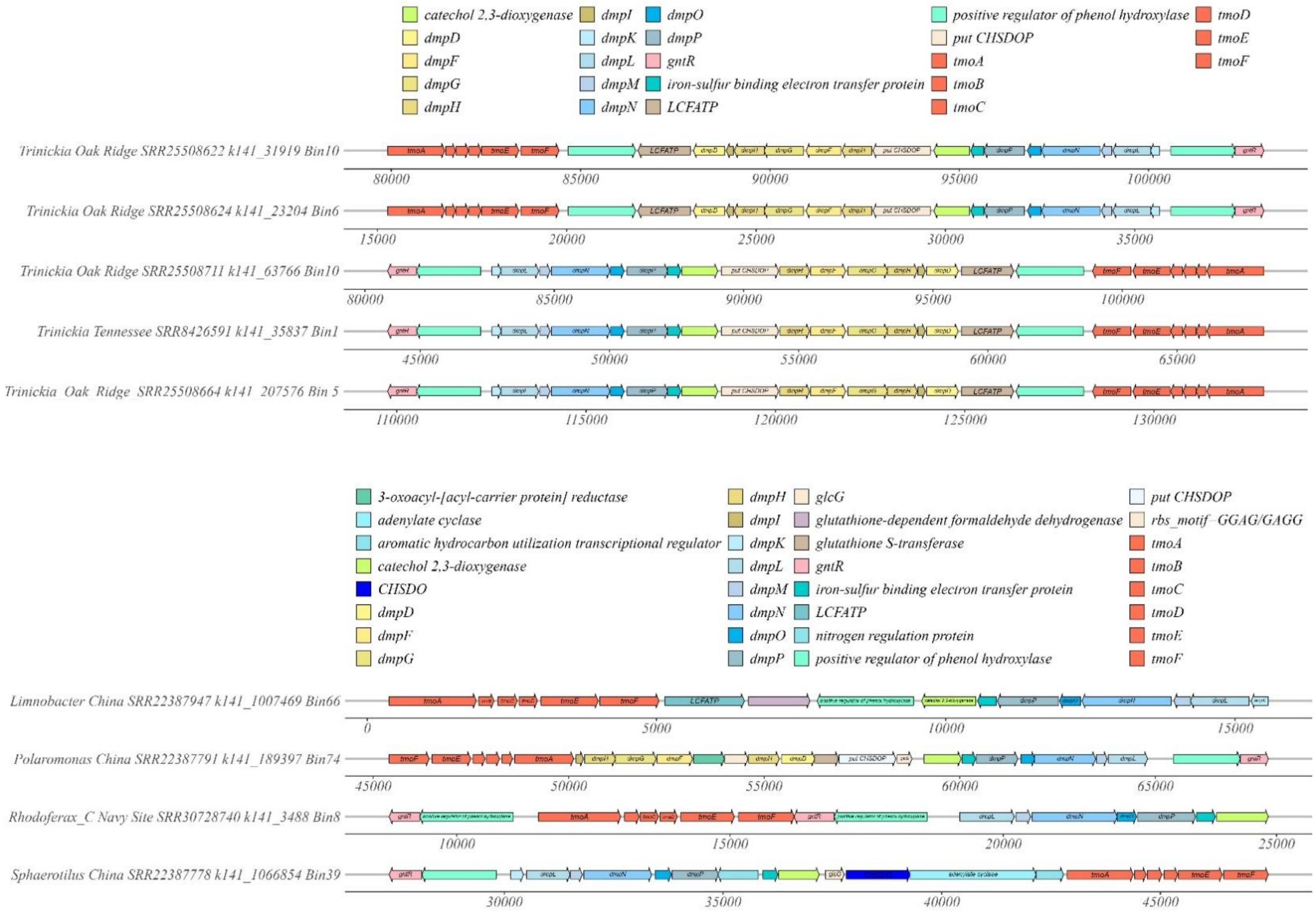

Supplementary Figure S10. Subunit length and arrangement of phenol monooxygenase operons in close proximity to a toluene monooxygenase operon. The operons contain catechol 2,3-dioxygenase, phenol hydroxylase assembly protein (*dmpK*), P1 oxygenase component (*dmpL*), P2 regulatory component (*dmpM*), P3 oxygenase component (*dmpN*), P4 oxygenase component (*dmpO*), FAD- and [2Fe-2S]-containing reductase component (*dmpP*), protein GlcG (*glcG*), regulatory protein GntR (*gntR*), irom-sulfur binding electron transfer protein, positive regulator of phenol hydroxylase, long-chain fatty acid transport protein (LCFATP), putative 5-carboxymethyl-2-hydroxymuconate semialdehyde dehydrogenase oxidoreductase (put CHSDOP).

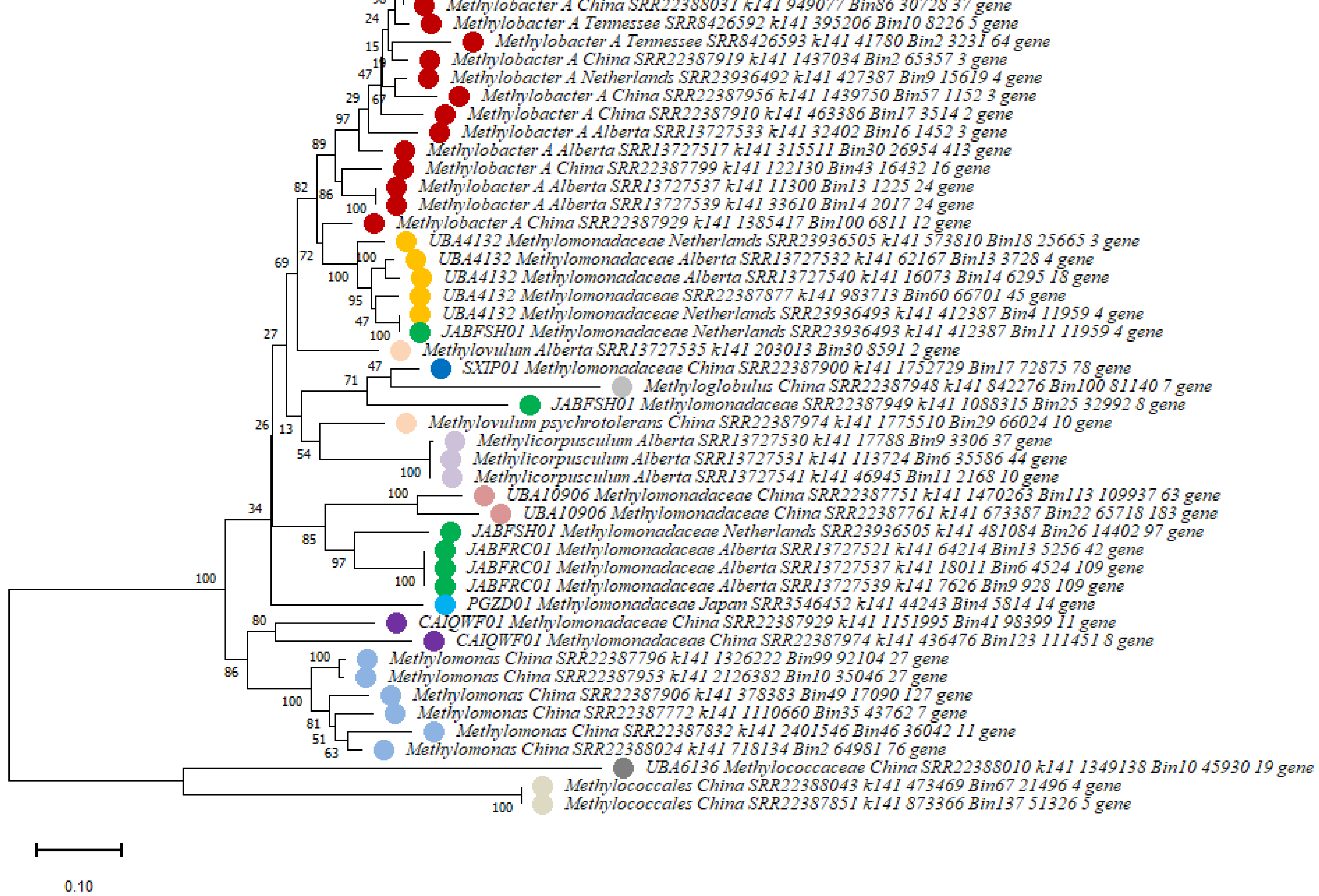

Supplementary Figure S11. Alignment of *pmoA* from MAGs containing the particulate ammonia/methane monooxygenase operon within the *Methylococcales* (*Gammaproteobacteria*). Different phylotypes are illustrated with different colors. The evolutionary history was inferred by using the Maximum Likelihood method and General Time Reversible model. The percentage of trees in which the associated taxa clustered together is shown next to the branches. Initial tree(s) for the heuristic search were obtained automatically by applying Neighbor-Join and BioNJ algorithms to a matrix of pairwise distances estimated using the Maximum Composite Likelihood (MCL) approach, and then selecting the topology with superior log likelihood value. The tree is drawn to scale, with branch lengths measured in the number of substitutions per site. This analysis involved 46 nucleotide sequences. There were a total of 750 positions in the final dataset. Evolutionary analyses were conducted in MEGA12.

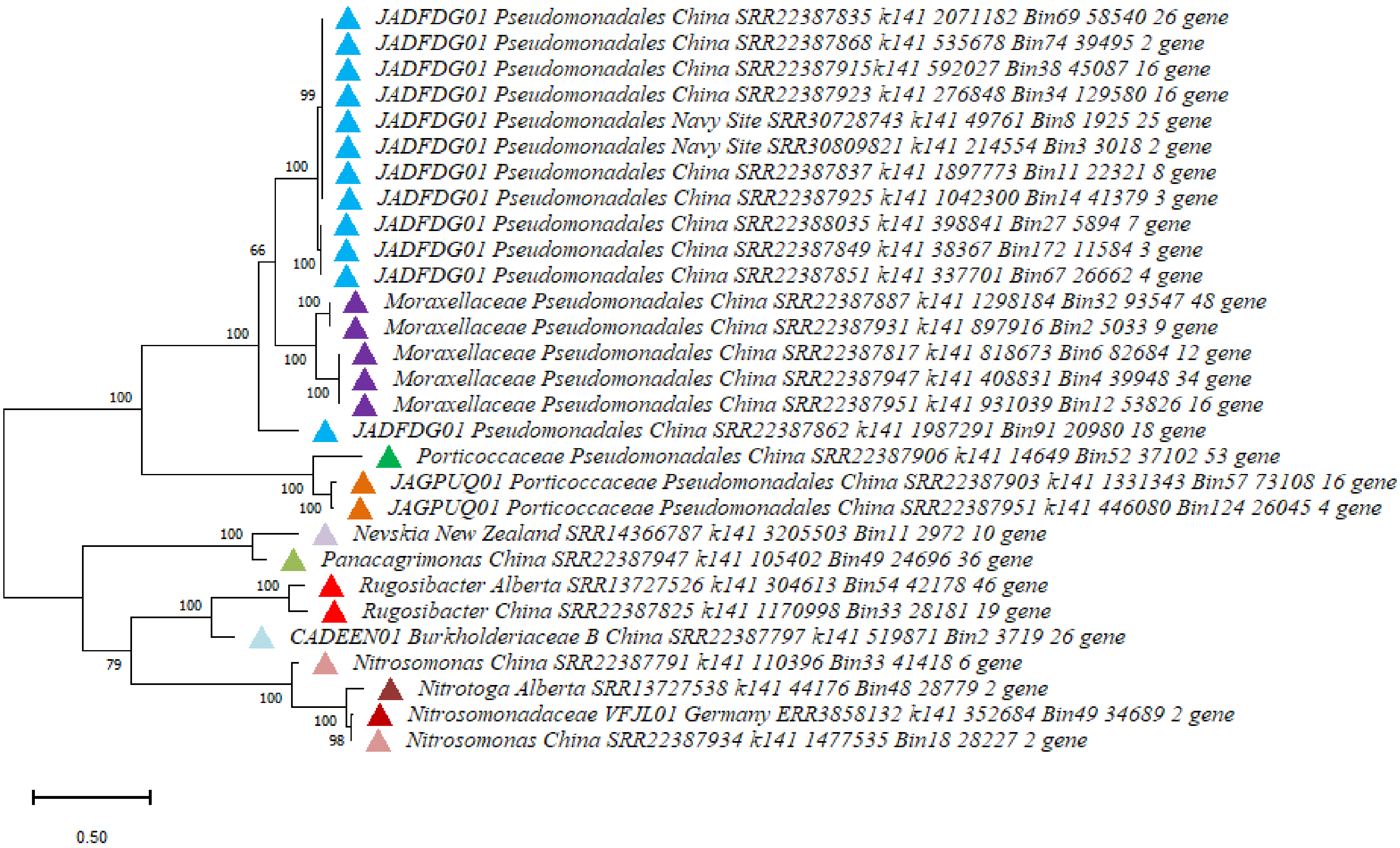

Supplementary Figure S12. Alignment of *pmoA* from MAGs containing the particulate ammonia/methane monooxygenase operon within the *Burkholderiales* and *Pseudomonadales* and *Nevskiales (Gammaproteobacteria)*. Different phylotypes are illustrated with different colors. The evolutionary history was inferred by using the Maximum Likelihood method and General Time Reversible model. The percentage of trees in which the associated taxa clustered together is shown next to the branches. Initial tree(s) for the heuristic search were obtained automatically by applying Neighbor-Join and BioNJ algorithms to a matrix of pairwise distances estimated using the Maximum Composite Likelihood (MCL) approach, and then selecting the topology with superior log likelihood value. The tree is drawn to scale, with branch lengths measured in the number of substitutions per site. This analysis involved 29 nucleotide sequences. There were a total of 879 positions in the final dataset. Evolutionary analyses were conducted in MEGA12.

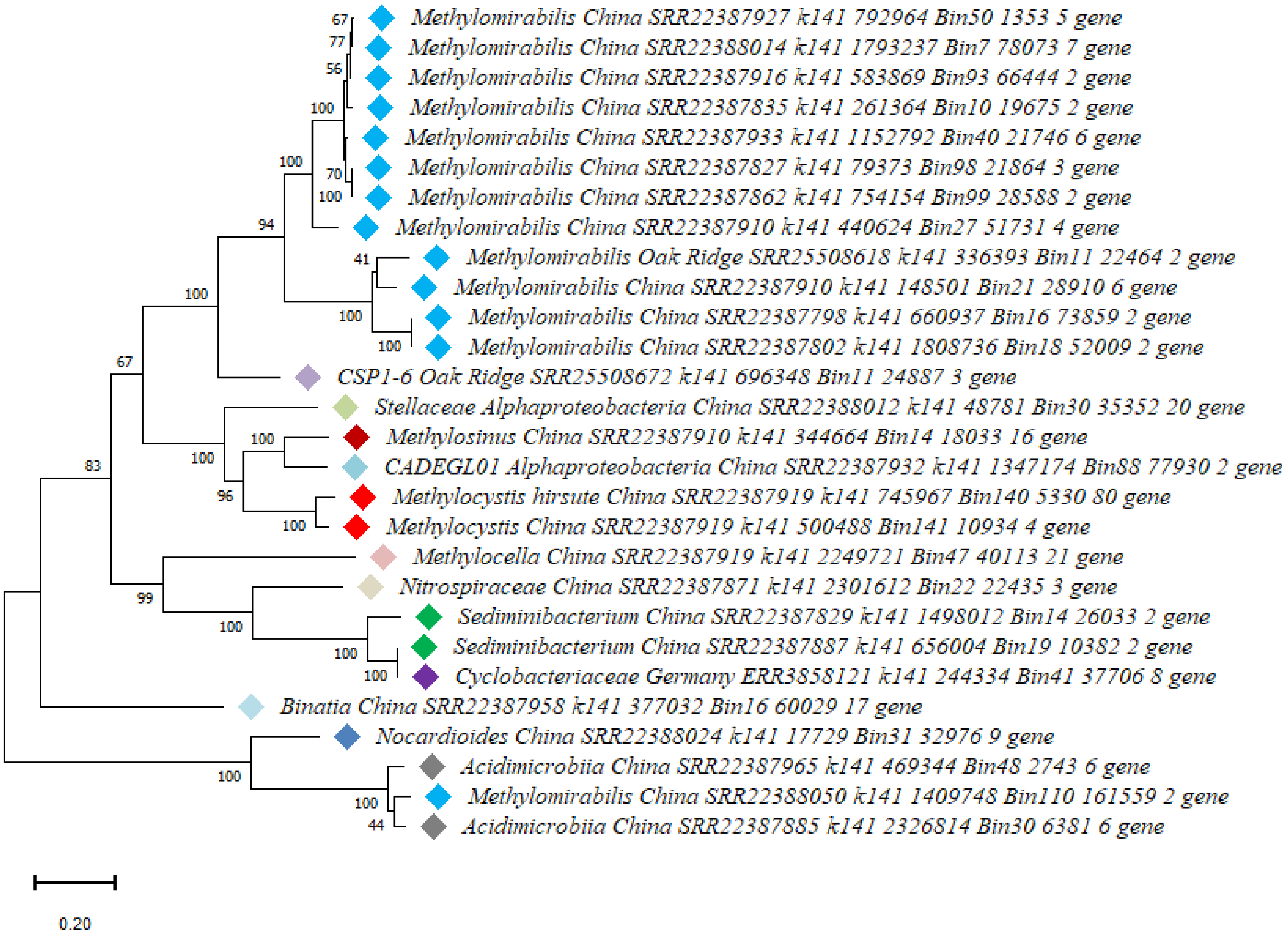

Supplementary Figure S13. Alignment of *pmoA* from MAGs containing the particulate ammonia/methane monooxygenase operon within the class *Alphaproteobacteria,* and phyla *Methylomirabilota, Bacteroidota, CSP1-3, Nitrospirota, Actinomycetota* and *Desulfobacterota_B.* Different phylotypes are illustrated with different colors. The evolutionary history was inferred by using the Maximum Likelihood method and General Time Reversible model. The percentage of trees in which the associated taxa clustered together is shown next to the branches. Initial tree(s) for the heuristic search were obtained automatically by applying Neighbor-Join and BioNJ algorithms to a matrix of pairwise distances estimated using the Maximum Composite Likelihood (MCL) approach, and then selecting the topology with superior log likelihood value. The tree is drawn to scale, with branch lengths measured in the number of substitutions per site. This analysis involved 28 nucleotide sequences. There were a total of 882 positions in the final dataset. Evolutionary analyses were conducted in MEGA12.

Table S1. MAGs containing four subunits of propane monooxygenase classifying within the phylum *Actinomycetota.*

		Class/Order	***Family***	***Genus***	***Species***
		Actinomycetia			
PRJNA699054 New Zealand	Bin.007.fastaSRR14366780	*Mycobacteriales*	*Mycobacteriaceae*	*Rhodococcus*	*Rhodococcus sp001647195*
PRJNA699054 New Zealand	Bin.025.fastaSRR14366780	*Mycobacteriales*	*Mycobacteriaceae*	*Mycobacterium*	*Mycobacterium sp023264755*
PRJNA1001011 Oak Ridge	Bin.015.fastaSRR25508637	*Mycobacteriales*	*Mycobacteriaceae*	*Mycobacterium*	
PRJNA1001011 Oak Ridge	Bin.047.fastaSRR25508634	*Mycobacteriales*	*Mycobacteriaceae*	*Mycobacterium*	
PRJNA858913 China	Bin.008.fastaSRR22387839	*Mycobacteriales*	*Mycobacteriaceae*	*Rhodococcus*	*Rhodococcus sp001647195*
PRJNA858913 China	Bin.024.fastaSRR22387839	*Mycobacteriales*	*Mycobacteriaceae*	*Rhodococcus*	*Rhodococcus qingshengii*
PRJNA858913 China	Bin.029.fastaSRR22387929	*Mycobacteriales*	*Mycobacteriaceae*	*Williamsia*	
PRJNA858913 China	Bin.020.fastaSRR22387962	*Mycobacteriales*	*Mycobacteriaceae*	*Rhodococcus*	*Rhodococcus opacus*
PRJNA858913 China	Bin.016.fastaSRR22387973	*Mycobacteriales*	*Mycobacteriaceae*	*Gordonia*	*Gordonia jacobaea*
PRJNA699054 New Zealand	Bin.029.fastaSRR14366791	*Mycobacteriales*	*Pseudonocardiaceae*	*GCA-003244245*	*GCA-003244245 sp023252195*
PRJNA700657 Alberta	Bin.005.fastaSRR13727533	*Propionibacteriales*	*Nocardioidaceae*	*Unclassified*	
		Rubrobacteria			
PRJNA858913 China	Bin.023.fastaSRR22387827	*Rubrobacterales*	*Rubrobacteraceae*	*Rubrobacter_A*	
PRJNA858913 China	Bin.108.fastaSRR22387827	*Rubrobacterales*	*Rubrobacteraceae*	*JACCWD01*	
		Thermoleophilia			
PRJNA700657 Alberta	Bin.020.fastaSRR13727533	*Solirubrobacterales*	*Solirubrobacteraceae*	*Patulibacter*	
PRJNA858913 China	Bin.127.fastaSRR22387974	*Solirubrobacterales*	*Solirubrobacteraceae*	*Palsa-465*	
PRJNA858913 China	Bin.058.fastaSRR22388024	*Solirubrobacterales*	*Solirubrobacteraceae*	*CADCVQ01*	
PRJNA858913 China	Bin.012.fastaSRR22388026	*Solirubrobacterales*	*Solirubrobacteraceae*	*Palsa-465*	
PRJNA858913 China	Bin.064.fastaSRR22388040	*Solirubrobacterales*	*Solirubrobacteraceae*	*Palsa-465*	

Table S2. MAGs containing four subunits of propane monooxygenase classifying within the phyla *Pseudomonadota* and *Chloroflexota*.

		Phylum/Class/Order	***Family***	***Genus***	***Species***
		Pseudomonadota /Alphaproteobacteria			
PRJNA700657 Alberta	Bin.022.fastaSRR13727533	*Rhizobiales*	*Hyphomicrobiaceae*	*JAKAXS01*	
PRJNA1162924 Navy Site	Bin.006.fastaSRR30728736	*Rhizobiales*	*Beijerinckiaceae*	*Methylocella*	
PRJNA1162924 Navy Site	Bin.013.fastaSRR30809810	*Rhizobiales*	*Beijerinckiaceae*	*Methylocella*	
PRJNA858913 China	Bin.038.fastaSRR22387819	*Rhizobiales*	*Xanthobacteraceae*	*Bradyrhizobium*	
PRJNA858913 China	Bin.009.fastaSRR22387878	*Rhizobiales*	*Xanthobacteraceae*	*Bradyrhizobium*	
PRJNA858913 China	Bin.085.fastaSRR22387808	*Rhizobiales*	*Rhizobiaceae*	*Mesorhizobium_A*	
PRJNA858913 China	Bin.003.fastaSRR22387879	*Acetobacterales*	*Acetobacteraceae*		
PRJNA858913 China	Bin.029.fastaSRR22388048	*Acetobacterales*	*Acetobacteraceae*		
PRJNA858913 China	Bin.041.fastaSRR22387936	*Rhodobacterales*	*Rhodobacteraceae*	*Gemmobacter*	*Gemmobacter aquatilis*
		Pseudomonadota /Gammaproteobacteria			
PRJNA858913 China	Bin.018.fastaSRR22387788	*Burkholderiales*	*Burkholderiaceae_B*	*Methylibium*	
PRJNA858913 China	Bin.030.fastaSRR22387859	*Burkholderiales*	*Burkholderiaceae_B*	*Methylibium*	
PRJNA858913 China	Bin.001.fastaSRR22387962	*Burkholderiales*	*Burkholderiaceae_B*	*Methylibium*	
PRJNA858913 China	Bin.061.fastaSRR22388023	*Burkholderiales*	*Rhodocyclaceae*	*Azoarcus*	
		Chloroflexota/ Ktedonobacteria			
PRJNA1001011 Oak Ridge	Bin.014.fastaSRR25508684	*Ktedonobacterales*	*Ktedonobacteraceae*	*CF-113*	
PRJNA1001011 Oak Ridge	Bin.011.fastaSRR25508678	*Ktedonobacterales*	*Ktedonobacteraceae*	*CF-114*	
PRJNA1001011 Oak Ridge	Bin.006.fastaSRR25508686	*Ktedonobacterales*	*Ktedonobacteraceae*	*CF-115*	

Table S3. MAGs containing soluble methane monooxygenase operons classifying within the *Gammaproteobacteria* and *Alphaproteobacteria.*

		Class/Order/Family	***Genus***	***Species***
		Gamma/ Methylococcales		
PRJNA513876 Tennessee	Bin.010.fastaSRR8426592	*Methylomonadaceae*	*Methylobacter_A*	*Methylobacter_A sp004299305*
PRJNA700657 Alberta	Bin.031.fastaSRR13727531	*Methylomonadaceae*	*Methylovulum*	
PRJNA700657 Alberta	Bin.003.fastaSRR13727532	*Methylomonadaceae*	*JABFRC01*	
PRJNA700657 Alberta	Bin.013.fastaSRR13727532	*Methylomonadaceae*	*UBA4133*	
PRJNA700657 Alberta	Bin.016.fastaSRR13727533	*Methylomonadaceae*	*Methylobacter_A*	
PRJNA700657 Alberta	Bin.006.fastaSRR13727537	*Methylomonadaceae*	*JABFRC01*	
PRJNA700657 Alberta	Bin.018.fastaSRR13727537	*Methylomonadaceae*	*UBA4132*	
PRJNA700657 Alberta	Bin.009.fastaSRR13727539	*Methylomonadaceae*	*JABFRC01*	
PRJNA700657 Alberta	Bin.001.fastaSRR13727540	*Methylomonadaceae*	*JABFRC01*	
PRJNA916093 Bangladesh	Bin.007.fastaSRR23060517	*Methylomonadaceae*	*SXIZ01*	
PRJNA916093 Bangladesh	Bin.016.fastaSRR23060517	*Methylomonadaceae*	*Methylomonas*	
PRJNA858913 China	Bin.113.fastaSRR22387751	*Methylomonadaceae*	*UBA10906*	
PRJNA858913 China	Bin.001.fastaSRR22387754	*Methylomonadaceae*	*UBA10906*	
PRJNA858913 China	Bin.022.fastaSRR22387761	*Methylomonadaceae*	*UBA10906*	
PRJNA858913 China	Bin.099.fastaSRR22387796	*Methylomonadaceae*	*Methylomonas*	
PRJNA858913 China	Bin.049.fastaSRR22387906	*Methylomonadaceae*	*Methylomonas*	
PRJNA858913 China	Bin.041.fastaSRR22387929	*Methylomonadaceae*	*CAIQWF01*	
PRJNA858913 China	Bin.010.fastaSRR22387953	*Methylomonadaceae*	*Methylomonas*	
PRJNA858913 China	Bin.057.fastaSRR22387956	*Methylomonadaceae*	*Methylobacter_A*	*Methylobacter_A tundripaludum_A*
		Gamma/ Burkholderiales		
PRJNA321556 Japan	Bin.054.fastaSRR3546449	*Methylophilaceae*	*UBA6140*	*UBA6140 sp002842435*
PRJNA321556 Japan	Bin.007.fastaSRR3546450	*Methylophilaceae*	*UBA6140*	*UBA6140 sp002842435*
PRJNA321556 Japan	Bin.025.fastaSRR3546451	*Methylophilaceae*	*UBA6140*	*UBA6140 sp002842435*
PRJNA321556 Japan	Bin.021.fastaSRR3546453	*Methylophilaceae*	*UBA6140*	*UBA6140 sp002842435*
PRJNA321556 Japan	Bin.030.fastaSRR3546454	*Methylophilaceae*	*UBA6140*	*UBA6140 sp002842435*
		Alpha/ Rhizobiales		
PRJNA1162924 Navy Site	Bin.006.fastaSRR30728736	*Beijerinckiaceae*	*Methylocella*	
PRJNA1162924 Navy Site	Bin.013.fastaSRR30809810	*Beijerinckiaceae*	*Methylocella*	

Table S4. MAGs containing particulate ammonia/particulate methane monooxygenase operons classifying within the *Methylococcales (Gammaproteobacteria).*

		Order	Family	Genus	Species
**PRJNA513876 Tennessee**	**Bin.010.fastaSRR8426592**	***Methylococcales***	***Methylomonadaceae***	***Methylobacter_A***	***Methylobacter_A sp004299305***
PRJNA513876 Tennessee	Bin.002.fastaSRR8426593	*Methylococcales*	*Methylomonadaceae*	*Methylobacter_A*	*Methylobacter_A sp004322395*
**PRJNA947390 Netherlands**	**Bin.009.fastaSRR23936492**	***Methylococcales***	***Methylomonadaceae***	***Methylobacter_A***	
**PRJNA700657 Alberta**	**Bin.030.fastaSRR13727517**	***Methylococcales***	***Methylomonadaceae***	***Methylobacter_A***	
PRJNA700657 Alberta	Bin.020.fastaSRR13727518	*Methylococcales*	*Methylomonadaceae*	*Methylobacter_A*	
PRJNA700657 Alberta	Bin.016.fastaSRR13727533	*Methylococcales*	*Methylomonadaceae*	*Methylobacter_A*	
PRJNA700657 Alberta	Bin.013.fastaSRR13727537	*Methylococcales*	*Methylomonadaceae*	*Methylobacter_A*	
PRJNA700657 Alberta	Bin.014.fastaSRR13727539	*Methylococcales*	*Methylomonadaceae*	*Methylobacter_A*	
PRJNA858913 China	Bin.043.fastaSRR22387799	*Methylococcales*	*Methylomonadaceae*	*Methylobacter_A*	*Methylobacter_A sp023227905*
**PRJNA858913 China**	**Bin.094.fastaSRR22387818**	***Methylococcales***	***Methylomonadaceae***	***Methylobacter_A***	
PRJNA858913 China	Bin.017.fastaSRR22387910	*Methylococcales*	*Methylomonadaceae*	*Methylobacter_A*	
PRJNA858913 China	Bin.002.fastaSRR22387919	*Methylococcales*	*Methylomonadaceae*	*Methylobacter_A*	
PRJNA858913 China	Bin.100.fastaSRR22387929	*Methylococcales*	*Methylomonadaceae*	*Methylobacter_A*	
**PRJNA858913 China**	**Bin.057.fastaSRR22387956**	***Methylococcales***	***Methylomonadaceae***	***Methylobacter_A***	***Methylobacter_A tundripaludum_A***
PRJNA858913 China	Bin.086.fastaSRR22388031	*Methylococcales*	*Methylomonadaceae*	*Methylobacter_A*	
**PRJNA947390 Netherlands**	**Bin.004.fastaSRR23936493**	***Methylococcales***	***Methylomonadaceae***	***UBA4132***	
PRJNA947390 Netherlands	Bin.018.fastaSRR23936505	*Methylococcales*	*Methylomonadaceae*	*UBA4132*	
**PRJNA700657 Alberta**	**Bin.013.fastaSRR13727532**	***Methylococcales***	***Methylomonadaceae***	***UBA4132***	
PRJNA700657 Alberta	Bin.014.fastaSRR13727540	*Methylococcales*	*Methylomonadaceae*	*UBA4132*	
**PRJNA858913 China**	**Bin.060.fastaSRR22387877**	***Methylococcales***	***Methylomonadaceae***	***UBA4132***	
**PRJNA947390 Netherlands**	**Bin.011.fastaSRR23936505**	***Methylococcales***	***Methylomonadaceae***	***JABFSH01***	
PRJNA947390 Netherlands	Bin.026.fastaSRR23936505	*Methylococcales*	*Methylomonadaceae*	*JABFSH01*	
**PRJNA700657 Alberta**	**Bin.013.fastaSRR137285121**	***Methylococcales***	***Methylomonadaceae***	***JABFRC01***	
PRJNA700657 Alberta	Bin.006.fastaSRR13727537	*Methylococcales*	*Methylomonadaceae*	*JABFRC01*	
PRJNA700657 Alberta	Bin.009.fastaSRR13727539	*Methylococcales*	*Methylomonadaceae*	*JABFRC01*	
**PRJNA858913 China**	**Bin.025.fastaSRR22387949**	***Methylococcales***	***Methylomonadaceae***	***JABFSH01***	
**PRJNA321556 Japan**	**Bin.004.fastaSRR3546452**	***Methylococcales***	***Methylomonadaceae***	***PGZD01***	*PGZD01 sp002843155*
**PRJNA858913 China**	**Bin.017.fastaSRR22387900**	***Methylococcales***	***Methylomonadaceae***	***SXIP01***	*SXIP01 sp005772825*
**PRJNA858913 China**	**Bin.041.fastaSRR22387929**	***Methylococcales***	***Methylomonadaceae***	***CAIQWF01***	
PRJNA858913 China	Bin.123.fastaSRR22387974	*Methylococcales*	*Methylomonadaceae*	*CAIQWF01*	
**PRJNA858913 China**	**Bin.113.fastaSRR22387751**	***Methylococcales***	***Methylomonadaceae***	***UBA10906***	
PRJNA858913 China	Bin.022.fastaSRR22387761	*Methylococcales*	*Methylomonadaceae*	*UBA10906*	
**PRJNA700657 Alberta**	**Bin.009.fastaSRR13727530**	***Methylococcales***	***Methylomonadaceae***	***Methylicorpusculum***	
PRJNA700657 Alberta	Bin.006.fastaSRR13727531	*Methylococcales*	*Methylomonadaceae*	*Methylicorpusculum*	
PRJNA700657 Alberta	Bin.011.fastaSRR13727541	*Methylococcales*	*Methylomonadaceae*	*Methylicorpusculum*	
PRJNA700657 Alberta	Bin.030.fastaSRR13727535	*Methylococcales*	*Methylomonadaceae*	*Methylovulum*	
**PRJNA858913 China**	**Bin.029.fastaSRR22387974**	***Methylococcales***	***Methylomonadaceae***	***Methylovulum***	***Methylovulum psychrotolerans***
**PRJNA858913 China**	**Bin.035.fastaSRR22387772**	***Methylococcales***	***Methylomonadaceae***	***Methylomonas***	

PRJNA858913 China	Bin.099.fastaSRR22387796	*Methylococcales*	*Methylomonadaceae*	*Methylomonas*	
PRJNA858913 China	Bin.046.fastaSRR22387832	*Methylococcales*	*Methylomonadaceae*	*Methylomonas*	
PRJNA858913 China	Bin.049.fastaSRR22387906	*Methylococcales*	*Methylomonadaceae*	*Methylomonas*	
PRJNA858913 China	Bin.010.fastaSRR22387953	*Methylococcales*	*Methylomonadaceae*	*Methylomonas*	
PRJNA858913 China	Bin.002.fastaSRR22388024	*Methylococcales*	*Methylomonadaceae*	*Methylomonas*	
**PRJNA858913 China**	**Bin.100.fastaSRR22387948**	***Methylococcales***	***Methylomonadaceae***	***Methyloglobulus***	
**PRJNA858913 China**	**Bin.010.fastaSRR22388010**	***Methylococcales***	***Methylococcaceae***	***UBA6136***	
PRJNA858913 China	Bin.067.fastaSRR22388043	*Methylococcales*			
PRJNA858913 China	Bin.137.fastaSRR22387851	*Methylococcales*			

Table S5. MAGs containing particulate ammonia/particulate methane monooxygenase operons classifying within the *Burkholderiales* and *Pseudomonadales* and *Nevskiales (Gammaproteobacteria)*.

		Order	Family	Genus	Species
**PRJEB36505 Germany**	**Bin.049.fastaERR3858132**	***Burkholderiales***	***Nitrosomonadaceae***	***VFJL01***	***VFJL01 sp919902515***
**PRJNA858913 China**	**Bin.033.fastaSRR22387791**	***Burkholderiales***	***Nitrosomonadaceae***	***Nitrosomonas***	***Nitrosomonas sp919902625***
PRJNA858913 China	Bin.018.fastaSRR22387934	*Burkholderiales*	*Nitrosomonadaceae*	*Nitrosomonas*	*Nitrosomonas sp919902625*
**PRJNA700657 Alberta**	**Bin.054.fastaSRR137275126**	***Burkholderiales***	***Rhodocyclaceae***	***Rugosibacter***	***Rugosibacter sp016235025***
**PRJNA858913 China**	**Bin.033.fastaSRR22387825**	***Burkholderiales***	***Rhodocyclaceae***	***Rugosibacter***	
**PRJNA700657 Alberta**	**Bin.048.fastaSRR13727538**	***Burkholderiales***	***Gallionellaceae***	***Nitrotoga***	***Nitrotoga sp003402375***
**PRJNA858913 China**	**Bin.002.fastaSRR22387797**	***Burkholderiales***	***Burkholderiaceae_B***	***CADEEN01***	
**PRJNA1162924 Navy Site**	**Bin.008.fastaSRR30728743**	***Pseudomonadales***	***JADFDG01***	***JADFDG01***	
PRJNA1162924 Navy Site	Bin.003.fastaSRR30809821	*Pseudomonadales*	*JADFDG01*	*JADFDG01*	
**PRJNA858913 China**	**Bin.029.fastaSRR22387785**	***Pseudomonadales***	***JADFDG01***	***JADFDG01***	
PRJNA858913 China	Bin.069.fastaSRR22387835	*Pseudomonadales*	*JADFDG01*	*JADFDG01*	
PRJNA858913 China	Bin.011.fastaSRR22387837	*Pseudomonadales*	*JADFDG01*	*JADFDG01*	
PRJNA858913 China	Bin.172.fastaSRR22387849	*Pseudomonadales*	*JADFDG01*	*JADFDG01*	
PRJNA858913 China	Bin.067.fastaSRR22387851	*Pseudomonadales*	*JADFDG01*	*JADFDG01*	
PRJNA858913 China	Bin.091.fastaSRR22387862	*Pseudomonadales*	*JADFDG01*	*JADFDG01*	
PRJNA858913 China	Bin.074.fastaSRR22387868	*Pseudomonadales*	*JADFDG01*	*JADFDG01*	
PRJNA858913 China	Bin.038.fastaSRR22387915	*Pseudomonadales*	*JADFDG01*	*JADFDG01*	
PRJNA858913 China	Bin.034.fastaSRR22387923	*Pseudomonadales*	*JADFDG01*	*JADFDG01*	
PRJNA858913 China	Bin.014.fastaSRR22387925	*Pseudomonadales*	*JADFDG01*	*JADFDG01*	
PRJNA858913 China	Bin.027.fastaSRR22388035	*Pseudomonadales*	*JADFDG01*	*JADFDG01*	
PRJNA858913 China	Bin.003.fastaSRR22387787	*Pseudomonadales*			
**PRJNA858913 China**	**Bin.039.fastaSRR22387782**	***Pseudomonadales***	***UBA3067***		
**PRJNA858913 China**	**Bin.058.fastaSRR22387903**	***Pseudomonadales***	***Porticoccaceae***	***JAGPUQ01***	***JAGPUQ01 sp016708925***
PRJNA858913 China	Bin.052.fastaSRR22387906	*Pseudomonadales*	*Porticoccaceae*		
PRJNA858913 China	Bin.124.fastaSRR22387951	*Pseudomonadales*	*Porticoccaceae*	*JAGPUQ01*	*JAGPUQ01 sp016708925*
**PRJNA858913 China**	**Bin.006.fastaSRR22387817**	***Pseudomonadales***	***Moraxellaceae***		
PRJNA858913 China	Bin.032.fastaSRR22387887	*Pseudomonadales*	*Moraxellaceae*		
PRJNA858913 China	Bin.004.fastaSRR22387947	*Pseudomonadales*	*Moraxellaceae*		
PRJNA858913 China	Bin.002.fastaSRR22387931	*Pseudomonadales*	*Moraxellaceae*		
PRJNA858913 China	Bin.012.fastaSRR22387951	*Pseudomonadales*	*Moraxellaceae*		
**PRJNA699054 New Zealand**	**Bin.011.fasta_SRR14366787**	***Nevskiales***	***Nevskiaceae***	***Nevskia***	***Nevskia sp023266035***
**PRJNA858913 China**	**Bin.049.fastaSRR22387947**	***Nevskiales***	***Nevskiaceae***	***Panacagrimonas***	

Table S6. MAGs containing particulate ammonia/particulate methane monooxygenase operons classifying within the class *Alphaproteobacteria,* and phyla *Methylomirabilota, Bacteroidota, CSP1-3, Nitrospirota, Actinomycetota* and *Desulfobacterota_B.*

		Class/Order	Family	Genus	Species
		Alphaproteobacteria			
**PRJNA858913 China**	**Bin.014.fastaSRR22387910**	***Rhizobiales***	***Beijerinckiaceae***	***Methylosinus***	
**PRJNA858913 China**	**Bin.140.fastaSRR22387919**	***Rhizobiales***	***Beijerinckiaceae***	***Methylocystis***	***Methylocystis hirsuta***
PRJNA858913 China	Bin.141.fastaSRR22387919	*Rhizobiales*	*Beijerinckiaceae*	*Methylocystis*	
**PRJNA858913 China**	**Bin.147.fastaSRR22387919**	***Rhizobiales***	***Beijerinckiaceae***	***Methylocella***	
**PRJNA858913 China**	**Bin.088.fastaSRR22387932**	***CADEGL01***	***CADEGL01***	***CADEGL01***	
**PRJNA858913 China**	**Bin.030.fastaSRR22388012**	***ATCC43930***	***Stellaceae***		
		Methylomirabilota/ Methylomirabilia			
**PRJNA1001011 Oak Ridge**	**Bin.011.fastaSRR25508618**	***Methylomirabilales***	***Methylomirabilaceae***	***Methylomirabilis***	
**PRJNA858913 China**	**Bin.040.fastaSRR22387783**	***Methylomirabilales***	***Methylomirabilaceae***	***Methylomirabilis***	
PRJNA858913 China	Bin.016.fastaSRR22387798	*Methylomirabilales*	*Methylomirabilaceae*	*Methylomirabilis*	
PRJNA858913 China	Bin.018.fastaSRR22387802	*Methylomirabilales*	*Methylomirabilaceae*	*Methylomirabilis*	
PRJNA858913 China	Bin.098.fastaSRR22387827	*Methylomirabilales*	*Methylomirabilaceae*	*Methylomirabilis*	
PRJNA858913 China	Bin.010.fastaSRR22387835	*Methylomirabilales*	*Methylomirabilaceae*	*Methylomirabilis*	
PRJNA858913 China	Bin.099.fastaSRR22387862	*Methylomirabilales*	*Methylomirabilaceae*	*Methylomirabilis*	
PRJNA858913 China	Bin.021.fastaSRR22387910	*Methylomirabilales*	*Methylomirabilaceae*	*Methylomirabilis*	
PRJNA858913 China	Bin.027.fastaSRR22387910	*Methylomirabilales*	*Methylomirabilaceae*	*Methylomirabilis*	
PRJNA858913 China	Bin.093.fastaSRR22387916	*Methylomirabilales*	*Methylomirabilaceae*	*Methylomirabilis*	
PRJNA858913 China	Bin.050.fastaSRR22387927	*Methylomirabilales*	*Methylomirabilaceae*	*Methylomirabilis*	
PRJNA858913 China	Bin.040.fastaSRR22387933	*Methylomirabilales*	*Methylomirabilaceae*	*Methylomirabilis*	
PRJNA858913 China	Bin.007.fastaSRR22388014	*Methylomirabilales*	*Methylomirabilaceae*	*Methylomirabilis*	
PRJNA858913 China	Bin.110.fastaSRR22388050	*Methylomirabilales*	*Methylomirabilaceae*	*Methylomirabilis*	*Methylomirabilis sp016207095*
		Bacteroidota/ Bacteroidia			
**PRJNA858913 China**	**Bin.014.fastaSRR22387829**	***Chitinophagales***	***Chitinophagaceae***	***Sediminibacterium***	***Sediminibacterium sp020403425***
PRJNA858913 China	Bin.019.fastaSRR22387887	*Chitinophagales*	*Chitinophagaceae*	*Sediminibacterium*	
**PRJEB36505 Germany**	**Bin.041.fastaERR3858121**	***Cytophagales***	***Cyclobacteriaceae***	***ELB16-189***	
		CSP1-3/ CSP1-4			
**PRJNA1001011 Oakridge**	**Bin.011.fastaSRR25508672**	***CSP1-5***	***CSP1-6***		
		Nitrospirota/ Nitrospiria			
**PRJNA858913 China**	**Bin.022.fastaSRR22387871**	***Nitrospirales***	***Nitrospiraceae***	***JACZQN01***	
		Actinomycetota/ Acidimicrobiia			
**PRJNA858913 China**	**Bin.030.fastaSRR22387885**	***IMCC26256***			
PRJNA858913 China	Bin.048.fastaSRR22387965	*IMCC26256*			
**PRJNA858913 China**	**Bin.031.fastaSRR22388024**	***Propionibacteriales***	***Nocardioidaceae***	***Nocardioides***	
		Desulfobacterota_B/ Binatia			
**PRJNA858913 China**	**Bin.016.fastaSRR22387958**	***Bin18***	***Bin18***	***JACQEL01***	

Table S7. MAGs containing toluene monooxygenase operons classifying within the family *Burkholderiaceae* *(Burkholderiales, Gammaproteobacteria).*

		**Family**	**Genus**	**Species**
PRJNA28802 Rifle CO	Bin.166.fastaSRR2090162	*Burkholderiaceae_B*	*Hydrogenophaga*	
PRJNA28802 Rifle CO	Bin.081.fastaSRR2090161	*Burkholderiaceae_B*	*Hydrogenophaga*	*Hydrogenophaga aromaticivorans*
PRJNA858913 China	Bin.034.fastaSRR22387899	*Burkholderiaceae_B*	*Hydrogenophaga*	*Hydrogenophaga pseudoflava*
PRJNA321556 Japan	Bin.007.fastaSRR3546449	*Burkholderiaceae_B*	*Hydrogenophaga*	*Hydrogenophaga sp002842215*
PRJNA321556 Japan	Bin.039.fastaSRR3546450	*Burkholderiaceae_B*	*Hydrogenophaga*	*Hydrogenophaga sp002842215*
PRJNA321556 Japan	Bin.030.fastaSRR3546451	*Burkholderiaceae_B*	*Hydrogenophaga*	*Hydrogenophaga sp002842215*
PRJNA512237 Ohio	Bin.053.fastaSRR8863435	*Burkholderiaceae*	*Rhodoferax*	*Rhodoferax sp008080595*
PRJNA1162924 Navy Site	Bin.003.fastaSRR30728740	*Burkholderiaceae_B*	*Rhodoferax*	*Rhodoferax sp019973615*
PRJNA1162924 Navy Site	Bin.013.fastaSRR30728742	*Burkholderiaceae_B*	*Rhodoferax*	*Rhodoferax sp019973615*
PRJNA1162924 Navy Site	Bin.015.fastaSRR30809814	*Burkholderiaceae_B*	*Rhodoferax*	*Rhodoferax sp019973615*
PRJNA1162924 Navy Site	Bin.005.fastaSRR30809820	*Burkholderiaceae_B*	*Rhodoferax*	*Rhodoferax sp019973615*
PRJNA1162924 Navy Site	Bin.008.fastaSRR30728740	*Burkholderiaceae_B*	*Rhodoferax_C*	*Unclassified*
PRJNA858913 China	Bin.083.fastaSRR22387975	*Burkholderiaceae_B*	*Rhodoferax*	*Rhodoferax sp012927445*
PRJNA858913 China	Bin.084.fastaSRR22387770	*Burkholderiaceae_B*	*Rhodoferax*	
PRJNA1162924 Navy Site	Bin.007.fastaSRR30809816	*Burkholderiaceae_B*	*Malikia*	
PRJNA1162924 Navy Site	Bin.011.fastaSRR30809817	*Burkholderiaceae_B*	*Malikia*	
PRJNA858913 China	Bin.039.fastaSRR22387778	*Burkholderiaceae_B*	*Sphaerotilus*	
PRJNA858913 China	Bin.074.fastaSRR22387791	*Burkholderiaceae_B*	*Polaromonas*	
PRJNA858913 China	Bin.052.fastaSRR22387947	*Burkholderiaceae_B*	*JAAFJR01*	
PRJNA858913 China	Bin.130.fastaSRR22387958	*Burkholderiaceae_B*	*Ramlibacter*	
PRJNA513876 Tennessee	Bin.001.fastaSRR8426591	*Burkholderiaceae*	*Trinickia*	*Trinickia sp004322045*
PRJNA1001011 Oak Ridge	Bin.001.fastaSRR25508605	*Burkholderiaceae*	*Trinickia*	*Trinickia sp004322045*
PRJNA1001011 Oak Ridge	Bin.010.fastaSRR25508622	*Burkholderiaceae*	*Trinickia*	*Trinickia sp004322045*
PRJNA1001011 Oak Ridge	Bin.006.fastaSRR25508624	*Burkholderiaceae*	*Trinickia*	*Trinickia sp004322045*
PRJNA1001011 Oak Ridge	Bin.005.fastaSRR25508664	*Burkholderiaceae*	*Trinickia*	*Trinickia sp004322045*
PRJNA1001011 Oak Ridge	Bin.010.fastaSRR25508711	*Burkholderiaceae*	*Trinickia*	*Trinickia sp004322045*
PRJNA1001011 Oak Ridge	Bin.017.fastaSRR25508712	*Burkholderiaceae*	*Trinickia*	*Trinickia sp004322045*
PRJNA858913 China	Bin.021.fastaSRR22388012	*Burkholderiaceae*	*Trinickia*	
PRJNA858913 China	Bin.066.fastaSRR22387947	*Burkholderiaceae*	*Limnobacter*	*Limnobacter sp018815245*
PRJNA858913 China	Bin.043.fastaSRR22388012	*Burkholderiaceae*	*LX47W*	*LX47W sp014042365*

Table S8. MAGs containing toluene monooxygenase operons classifying within the *Rhodocyclaceae, Nevskiaceae (Gammaproteobacteria) and Alphaproteobacteria.*

		Order	***Family***	***Genus***	***Species***
PRJNA512237 Ohio	Bin.031.fastaSRR8863435	*Burkholderiales*	*Rhodocyclaceae*	*Azonexus*	*Azonexus sp008080565*
PRJNA321556 Japan	Bin.044.fastaSRR3546449	*Burkholderiales*	*Rhodocyclaceae*	*Azonexus*	*Azonexus sp002840785*
PRJNA321556 Japan	Bin.023.fastaSRR3546450	*Burkholderiales*	*Rhodocyclaceae*	*Azonexus*	*Azonexus sp002840785*
PRJNA321556 Japan	Bin.038.fastaSRR3546453	*Burkholderiales*	*Rhodocyclaceae*	*Azonexus*	*Azonexus sp002842145*
PRJNA1162924 Navy Site	Bin.016.fastaSRR30728740	*Burkholderiales*	*Rhodocyclaceae*	*Azonexus*	
PRJNA1162924 Navy Site	Bin.001.fastaSRR30809814	*Burkholderiales*	*Rhodocyclaceae*	*Azonexus*	
PRJNA279923 Sweden	Bin.008.fastaSRR10598173	*Burkholderiales*	*Rhodocyclaceae*	*Azonexus*	*Azonexus sp018828385*
PRJNA279923 Sweden	Bin.019.fastaSRR10598174	*Burkholderiales*	*Rhodocyclaceae*	*Azonexus*	*Azonexus sp018828385*
PRJNA858913 China	Bin.057.fastaSRR22387794	*Burkholderiales*	*Rhodocyclaceae*	*Azonexus*	*Azonexus sp008080565*
PRJNA858913 China	Bin.004.fastaSRR22387795	*Burkholderiales*	*Rhodocyclaceae*	*Azonexus*	*Azonexus sp008080565*
PRJNA858913 China	Bin.008.fastaSRR22387801	*Burkholderiales*	*Rhodocyclaceae*	*Azonexus*	*Azonexus sp008080565*
PRJNA858913 China	Bin.015.fastaSRR22387833	*Burkholderiales*	*Rhodocyclaceae*	*Azonexus*	*Azonexus sp008080565*
PRJNA858913 China	Bin.036.fastaSRR22387860	*Burkholderiales*	*Rhodocyclaceae*	*Azonexus*	*Azonexus sp008080565*
PRJNA858913 China	Bin.109.fastaSRR22387871	*Burkholderiales*	*Rhodocyclaceae*	*Azonexus*	*Azonexus sp008080565*
PRJNA858913 China	Bin.013.fastaSRR22387971	*Burkholderiales*	*Rhodocyclaceae*	*Azonexus*	*Azonexus sp008080565*
PRJNA858913 China	Bin.040.fastaSRR22387858	*Burkholderiales*	*Rhodocyclaceae*	*Azonexus*	
PRJNA858913 China	Bin.035.fastaSRR22387866	*Burkholderiales*	*Rhodocyclaceae*	*Azonexus*	
PRJNA321556 Japan	Bin.002.fastaSRR3546454	*Burkholderiales*	*Rhodocyclaceae*	*Rugosibacter*	*Rugosibacter sp002842395*
PRJNA700657 Alberta	Bin.054.fastaSRR137275126	*Burkholderiales*	*Rhodocyclaceae*	*Rugosibacter*	*Rugosibacter sp016235025*
PRJNA858913 China	Bin.059.fastaSRR22388015	*Burkholderiales*	*Rhodocyclaceae*	*Rugosibacter*	
PRJNA858913 China	Bin.046.fastaSRR22387879	*Burkholderiales*	*Rhodocyclaceae*	*Zoogloea*	*Zoogloea sp002028455*
PRJNA858913 China	Bin.015.fastaSRR22387993	*Burkholderiales*	*Rhodocyclaceae*	*Zoogloea*	
PRJNA858913 China	Bin.053.fastaSRR22387820	*Burkholderiales*	*Rhodocyclaceae*		
PRJNA699054 New Zealand	Bin.011.fastaSRR14366787	*Nevskiales*	*Nevskiaceae*	*Nevskia*	*Nevskia sp023266035*
		Alpha			
PRJNA1162924 Navy Site	Bin.028.fastaSRR30728738	*Rhizobiales*	*Ancalomicrobiaceae*	*Pinisolibacter*	
PRJNA858913 China	Bin.002.fastaSRR22387879	*ATCC43930*	*Stellaceae*	*CAIXXC01*	

Table S9. MAGs containing toluene monooxygenase operons classifying within the family *Burkholderiaceae* *(Burkholderiales, Gammaproteobacteria)*.

		Family	Genus	Species
PRJNA321556 Japan	Bin.007.fastaSRR3546449	*Burkholderiaceae_B*	*Hydrogenophaga*	*Hydrogenophaga sp002842215*
PRJNA321556 Japan	Bin.039.fastaSRR3546450	*Burkholderiaceae_B*	*Hydrogenophaga*	*Hydrogenophaga sp002842215*
PRJNA321556 Japan	Bin.030.fastaSRR3546451	*Burkholderiaceae_B*	*Hydrogenophaga*	*Hydrogenophaga sp002842215*
PRJNA512237 Ohio	Bin.053.fastaSRR8863435	*Burkholderiaceae*	*Rhodoferax*	*Rhodoferax sp008080595*
PRJNA1162924 Navy Site	Bin.003.fastaSRR30728740	*Burkholderiaceae_B*	*Rhodoferax*	*Rhodoferax sp019973615*
PRJNA1162924 Navy Site	Bin.013.fastaSRR30728742	*Burkholderiaceae_B*	*Rhodoferax*	*Rhodoferax sp019973615*
PRJNA1162924 Navy Site	Bin.015.fastaSRR30809814	*Burkholderiaceae_B*	*Rhodoferax*	*Rhodoferax sp019973615*
PRJNA1162924 Navy Site	Bin.005.fastaSRR30809820	*Burkholderiaceae_B*	*Rhodoferax*	*Rhodoferax sp019973615*
PRJNA1162924 Navy Site	Bin.008.fastaSRR30728740	*Burkholderiaceae_B*	*Rhodoferax_C*	*Unclassified*
PRJNA858913 China	Bin.083.fastaSRR22387975	*Burkholderiaceae_B*	*Rhodoferax*	*Rhodoferax sp012927445*
PRJNA858913 China	Bin.039.fastaSRR22387778	*Burkholderiaceae_B*	*Sphaerotilus*	
PRJNA858913 China	Bin.074.fastaSRR22387791	*Burkholderiaceae_B*	*Polaromonas*	
PRJNA858913 China	Bin.130.fastaSRR22387958	*Burkholderiaceae_B*	*Ramlibacter*	
PRJNA513876 Tennessee	Bin.001.fastaSRR8426591	*Burkholderiaceae*	*Trinickia*	*Trinickia sp004322045*
PRJNA1001011 Oak Ridge	Bin.010.fastaSRR25508622	*Burkholderiaceae*	*Trinickia*	*Trinickia sp004322045*
PRJNA1001011 Oak Ridge	Bin.006.fastaSRR25508624	*Burkholderiaceae*	*Trinickia*	*Trinickia sp004322045*
PRJNA1001011 Oak Ridge	Bin.005.fastaSRR25508664	*Burkholderiaceae*	*Trinickia*	*Trinickia sp004322045*
PRJNA1001011 Oak Ridge	Bin.010.fastaSRR25508711	*Burkholderiaceae*	*Trinickia*	*Trinickia sp004322045*
PRJNA858913 China	Bin.021.fastaSRR22388012	*Burkholderiaceae*	*Trinickia*	
PRJNA858913 China	Bin.066.fastaSRR22387947	*Burkholderiaceae*	*Limnobacter*	*Limnobacter sp018815245*

Table S10. MAGs containing both toluene monooxygenase and phenol monooxygenase operons within the *Rhodocyclaceae.*

		Order	*Family*	*Genus*	*Species*
PRJNA512237 Ohio	Bin.031.fastaSRR8863435	*Burkholderiales*	*Rhodocyclaceae*	*Azonexus*	*Azonexus sp008080565*
PRJNA321556 Japan	Bin.044.fastaSRR3546449	*Burkholderiales*	*Rhodocyclaceae*	*Azonexus*	*Azonexus sp002840785*
PRJNA321556 Japan	Bin.023.fastaSRR3546450	*Burkholderiales*	*Rhodocyclaceae*	*Azonexus*	*Azonexus sp002840785*
PRJNA321556 Japan	Bin.038.fastaSRR3546453	*Burkholderiales*	*Rhodocyclaceae*	*Azonexus*	*Azonexus sp002842145*
PRJNA1162924 Navy Site	Bin.016.fastaSRR30728740	*Burkholderiales*	*Rhodocyclaceae*	*Azonexus*	
PRJNA279923 Sweden	Bin.008.fastaSRR10598173	*Burkholderiales*	*Rhodocyclaceae*	*Azonexus*	*Azonexus sp018828385*
PRJNA279923 Sweden	Bin.019.fastaSRR10598174	*Burkholderiales*	*Rhodocyclaceae*	*Azonexus*	*Azonexus sp018828385*
PRJNA858913 China	Bin.008.fastaSRR22387801	*Burkholderiales*	*Rhodocyclaceae*	*Azonexus*	*Azonexus sp008080565*
PRJNA858913 China	Bin.015.fastaSRR22387833	*Burkholderiales*	*Rhodocyclaceae*	*Azonexus*	*Azonexus sp008080565*
PRJNA858913 China	Bin.036.fastaSRR22387860	*Burkholderiales*	*Rhodocyclaceae*	*Azonexus*	*Azonexus sp008080565*
PRJNA858913 China	Bin.109.fastaSRR22387871	*Burkholderiales*	*Rhodocyclaceae*	*Azonexus*	*Azonexus sp008080565*
PRJNA858913 China	Bin.040.fastaSRR22387858	*Burkholderiales*	*Rhodocyclaceae*	*Azonexus*	
PRJNA858913 China	Bin.046.fastaSRR22387879	*Burkholderiales*	*Rhodocyclaceae*	*Zoogloea*	*Zoogloea sp002028455*
PRJNA858913 China	Bin.015.fastaSRR22387993	*Burkholderiales*	*Rhodocyclaceae*	*Zoogloea*	

References

1. Anantharaman, K.; Brown, C.T.; Burstein, D.; Castelle, C.J.; Probst, A.J.; Thomas, B.C.; Williams, K.H.; Banfield, J.F. Analysis of five complete genome sequences for members of the class Peribacteria in the recently recognized *Peregrinibacteria* bacterial phylum. *PeerJ* **2016**, *4*, e1607, doi:10.7717/peerj.1607.
2. Anantharaman, K.; Brown, C.T.; Hug, L.A.; Sharon, I.; Castelle, C.J.; Probst, A.J.; Thomas, B.C.; Singh, A.; Wilkins, M.J.; Karaoz, U.; et al. Thousands of microbial genomes shed light on interconnected biogeochemical processes in an aquifer system. *Nat Commun* **2016**, *7*, 13219, doi:10.1038/ncomms13219.
3. Zaremba-Niedzwiedzka, K.; Caceres, E.F.; Saw, J.H.; Backstrom, D.; Juzokaite, L.; Vancaester, E.; Seitz, K.W.; Anantharaman, K.; Starnawski, P.; Kjeldsen, K.U.; et al. Asgard archaea illuminate the origin of eukaryotic cellular complexity. *Nature* **2017**, *541*, 353-358, doi:10.1038/nature21031.
4. Danczak, R.E.; Johnston, M.D.; Kenah, C.; Slattery, M.; Wilkins, M.J. Capability for arsenic mobilization in groundwater is distributed across broad phylogenetic lineages. *PLoS One* **2019**, *6*, 14(19):e0221694.
5. Mosley, O.E.; Gios, E.; Weaver, L.; Close, M.; Daughney, C.; van der Raaij, R.; Martindale, H.; Handley, K.M. Metabolic Diversity and Aero-Tolerance in Anammox Bacteria from Geochemically Distinct Aquifers. *mSystems* **2022**, *7*, e0125521, doi:10.1128/msystems.01255-21.
6. Tian, R.; Ning, D.; He, Z.; Zhang, P.; Spencer, S.J.; Gao, S.; Shi, W.; Wu, L.; Zhang, Y.; Yang, Y.; et al. Small and mighty: adaptation of superphylum Patescibacteria to groundwater environment drives their genome simplicity. *Microbiome* **2020**, *8*, 51, doi:10.1186/s40168-020-00825-w.
7. Lui, L.M.; Nielsen, T.N.; Smith, H.J.; Chandonia, J.M.; Kuehl, J.V.; Song, F.; Sczesnak, A.; Hendrickson, A.; Hazen, T.C.; Fields, M.W.; et al. Sediment and groundwater metagenomes from subsurface microbial communities from the Oak Ridge National Laboratory Oak Ridge Reservation, Oak Ridge, Tennessee, USA. *Microbiol Resour Announc* **2025**, e0001425, doi:10.1128/mra.00014-25.
8. Goff, J.L.; Lui, L.M.; Nielsen, T.N.; Poole, F.L.; Smith, H.J.; Walker, K.F.; Hazen, T.C.; Fields, M.W.; Arkin, A.P.; Adams, M.W.W. Mixed waste contamination selects for a mobile genetic element population enriched in multiple heavy metal resistance genes. *ISME Commun* **2024**, *4*, ycae064, doi:10.1093/ismeco/ycae064.
9. Hernsdorf, A.W.; Amano, Y.; Miyakawa, K.; Ise, K.; Suzuki, Y.; Anantharaman, K.; Probst, A.; Burstein, D.; Thomas, B.C.; Banfield, J.F. Potential for microbial H(2) and metal transformations associated with novel bacteria and archaea in deep terrestrial subsurface sediments. *ISME J* **2017**, *11*, 1915-1929, doi:10.1038/ismej.2017.39.
10. Ruff, S.E.; Humez, P.; de Angelis, I.H.; Diao, M.; Nightingale, M.; Cho, S.; Connors, L.; Kuloyo, O.O.; Seltzer, A.; Bowman, S.; et al. Hydrogen and dark oxygen drive microbial productivity in diverse groundwater ecosystems. *Nat Commun* **2023**, *14*, 3194, doi:10.1038/s41467-023-38523-4.
11. Dang, H.; Cupples, A.M. Diversity and abundance of the functional genes and bacteria associated with RDX degradation at a contaminated site pre- and post-biostimulation. *Appl Microbiol Biotechnol* **2021**, *105*, 6463-6475, doi:10.1007/s00253-021-11457-x.

12. Wu, X.; Holmfeldt, K.; Hubalek, V.; Lundin, D.; Astrom, M.; Bertilsson, S.; Dopson, M. Microbial metagenomes from three aquifers in the Fennoscandian shield terrestrial deep biosphere reveal metabolic partitioning among populations. *ISME J* **2016**, *10*, 1192-1203, doi:10.1038/ismej.2015.185.
13. Wu, Z.; Liu, T.; Chen, Q.; Chen, T.; Hu, J.; Sun, L.; Wang, B.; Li, W.; Ni, J. Unveiling the unknown viral world in groundwater. *Nat Commun* **2024**, *15*, 6788, doi:10.1038/s41467-024-51230-y.
14. Diba, F.; Hoque, M.N.; Rahman, M.S.; Haque, F.; Rahman, K.M.J.; Moniruzzaman, M.; Khan, M.; Hossain, M.A.; Sultana, M. Metagenomic and culture-dependent approaches unveil active microbial community and novel functional genes involved in arsenic mobilization and detoxification in groundwater. *BMC Microbiol* **2023**, *23*, 241, doi:10.1186/s12866-023-02980-0.
15. Chaudhari, N.M.; Overholt, W.A.; Figueroa-Gonzalez, P.A.; Taubert, M.; Bornemann, T.L.V.; Probst, A.J.; Holzer, M.; Marz, M.; Kusel, K. The economical lifestyle of CPR bacteria in groundwater allows little preference for environmental drivers. *Environ Microbiome* **2021**, *16*, 24, doi:10.1186/s40793-021-00395-w.
16. Hauptfeld, E.; Pappas, N.; van Iwaarden, S.; Snoek, B.L.; Aldas-Vargas, A.; Dutilh, B.E.; von Meijenfeldt, F.A.B. Integrating taxonomic signals from MAGs and contigs improves read annotation and taxonomic profiling of metagenomes. *Nat Commun* **2024**, *15*, 3373, doi:10.1038/s41467-024-47155-1.
